# Dietary Modulation of CYP3A4 and Its Impact on Statins and Antidiabetic Drugs: A Narrative Review

**DOI:** 10.3390/ph18091351

**Published:** 2025-09-09

**Authors:** Manuel Hernández-Lorca, Isabel M. Timón, Pura Ballester, Paula Henarejos-Escudero, Ana María García-Muñoz, Desirée Victoria-Montesinos, Pablo Barcina-Pérez

**Affiliations:** Faculty of Pharmacy and Nutrition, UCAM Universidad Católica de Murcia, 30107 Murcia, Spain; mhernandez2@ucam.edu (M.H.-L.); imtimon@ucam.edu (I.M.T.); dvictoria@ucam.edu (D.V.-M.); pbarcina@ucam.edu (P.B.-P.)

**Keywords:** CYP3A4, drug metabolism, food–drug interaction, statins, antidiabetic agents, polyphenols, herbal supplements, pharmacokinetics

## Abstract

Cytochrome P450 3A4 (CYP3A4) is a key enzyme involved in the metabolism of nearly half of all clinically used drugs, including widely prescribed statins and antidiabetic agents. Dietary constituents can modulate CYP3A4 expression and activity through various mechanisms, thereby altering drug pharmacokinetics and potentially leading to therapeutic failure or toxicity. This narrative review compiles current evidence on dietary modulation of CYP3A4, with a particular focus on pharmacological and clinical implications for lipid-lowering and glucose-lowering drugs. Literature was identified through a comprehensive search in PubMed, Scopus, and Web of Science, including preclinical and clinical studies addressing food–drug interactions involving CYP3A4 substrates. Numerous dietary compounds, such as citrus furanocoumarins, polyphenols, herbal extracts, and vitamins, act as CYP3A4 inhibitors or inducers through competitive, mechanism-based, or nuclear receptor-mediated pathways. Specific examples include simvastatin, atorvastatin, repaglinide, and saxagliptin, whose systemic exposure can be significantly altered by dietary factors. Moreover, interindividual variability in CYP3A4 activity may be shaped by genetic polymorphisms, microbiota-derived metabolites, and epigenetic regulation, further influencing drug response. Understanding these interactions is crucial, especially in polymedicated patients or those receiving drugs with a narrow therapeutic index. Clinicians should remain aware of potential CYP3A4-related food–drug interactions and consider dietary habits and supplement use in therapeutic decision-making. Future research should aim to integrate pharmacogenomics, gut microbiome profiling, and personalized nutrition in order to improve the prediction and prevention of clinically significant interactions.

## 1. Introduction

Cytochrome P450 3A4 (CYP3A4) is a key enzyme in xenobiotic metabolism, implicated in the clearance of a substantial portion of approved pharmaceuticals [1,2]. Its broad substrate specificity and tissue distribution make it highly influential in determining drug pharmacokinetics and systemic exposure, particularly for medications such as lipid-lowering and glucose-lowering agents [3,4,5].

Among non-genetic factors, dietary intake has emerged as a relevant modulator of CYP3A4 activity. Several foods and bioactive compounds—such as grapefruit juice (GFJ)—are known to interact with this enzyme, altering drug bioavailability and therapeutic outcomes [6,7,8]. Similarly, botanical products like *Hypericum perforatum* have been shown to induce CYP3A4 expression through pregnane X receptor (PXR) activation, with clinically significant consequences [9,10,11].

Recent studies have expanded our understanding of dietary modulation of CYP3A4. For instance, red beetroot extract, rich in betanin, has been shown to inhibit CYP3A4 in a dose-dependent manner [12]. Similarly, aqueous extracts of Oolong tea were found to induce CYP3A expression in both hepatic and intestinal tissues in mice [13]. Moreover, new flavonoid compounds have been identified as potential CYP3A4 inhibitors, although their clinical relevance depends on their bioavailability [14,15].

These interactions are particularly relevant from a pharmacogenetic and toxicological perspective. Genetic variants in CYP3A4 and its regulatory pathways can modify individual susceptibility to diet–drug interactions, while exaggerated exposure or inadequate drug levels, driven by dietary modulation, particularly in the case of drugs with a narrow therapeutic index such as certain statins or glucose-lowering agents.

This narrative review aims to synthesize the current evidence on how foods, nutrients, and bioactive dietary compounds modulate CYP3A4 expression and activity, and to discuss the resulting pharmacokinetic and clinical implications for commonly prescribed statins and antidiabetic medications.

## 2. Methods

A literature search was conducted in PubMed, Scopus, and Web of Science to identify relevant studies published up to July 2025. The search was limited to peer-reviewed articles written in English. Keywords and their combinations included “CYP3A4,” “cytochrome P450,” “food–drug interaction,” and the names of commonly used statins and antidiabetic drugs. Both preclinical (in vitro and in vivo) and clinical studies, as well as recent narrative and systematic reviews, were considered if they addressed dietary influences on CYP3A4-mediated drug metabolism and clinical relevance. Conference abstracts, non-English publications, duplicate records, and studies not directly related to CYP3A4 modulation by dietary components were excluded. The selected literature was synthesized narratively to summarize current knowledge, pharmacokinetic and pharmacogenetic mechanisms, and implications for clinical practice.

## 3. Overview of CYP3A4

CYP3A4 is one of the most relevant drug-metabolizing enzymes in humans, belonging to the CYP3A subfamily, which participates in the metabolism of more than 30% [16] of currently marketed drugs, with some estimates reaching 45–60% [17]. Its broad substrate specificity, dual localization in the liver and intestine, and marked interindividual variability explain its central role in pharmacokinetics and in drug–drug and diet–drug interactions involving various treatments, including statins and antidiabetic agents.

CYP3A4 belongs to the cytochrome P450 (CYP450) superfamily, a group of membrane-bound hemoproteins that function as monooxygenases and are named for their characteristic absorption peak at 450 nm when reduced and bound to carbon monoxide [18]. The human CYP3A subfamily includes four isoforms—CYP3A4, CYP3A5, CYP3A7, and CYP3A43—whose genes are located on chromosome 7. Among these, CYP3A4 is the most abundant in the adult liver, whereas CYP3A7 predominates during the fetal and neonatal periods. Besides metabolizing xenobiotics, CYP3A4 also processes endogenous compounds, including steroid hormones, bile acids, and vitamin D [2,18].

The enzyme is mainly expressed in hepatocytes and enterocytes of the proximal small intestine, contributing to first-pass metabolism in both tissues [8,18]. In the liver, CYP3A4 accounts for about 30% of total hepatic CYP content, while in the intestine it is present predominantly in the duodenum and jejunum. This dual localization forms a “double metabolic barrier” that limits the oral bioavailability of many lipophilic drugs, such as simvastatin, atorvastatin, and repaglinide [8].

CYP3A4 exhibits remarkable substrate promiscuity due to its large and flexible active site, allowing it to metabolize a diverse range of compounds [19]. Clinically important substrates include lipophilic statins like simvastatin, atorvastatin, and lovastatin, whereas pravastatin and rosuvastatin are metabolized by other enzymes such as CYP2C9. Among oral antidiabetic drugs, pioglitazone, saxagliptin, and repaglinide are also processed via CYP3A4-mediated pathways. The enzyme predominantly catalyzes oxidation reactions, such as hydroxylation and dealkylation [2].

Importantly, CYP3A4 activity exhibits marked interindividual variability, with differences of up to 20–40-fold observed between patients [20]. Genetic factors contribute to this variability, although functional polymorphisms are less frequent compared to other isoenzymes. Variants such as CYP3A4*22* reduce enzyme expression, whereas CYP3A41B may increase it [21]. Factors such as sex, age, hormonal status, diet, smoking, supplements, and concomitant medications further modulate CYP3A4 activity. This wide variability helps explain divergent drug responses among patients receiving the same dosage of a CYP3A4 substrate.

The dual expression sites, broad substrate range, and high variability in activity have critical clinical implications. CYP3A4-mediated metabolism can decrease the oral bioavailability of drugs through first-pass clearance, while inhibition of the enzyme may result in elevated systemic drug concentrations and potential toxicity. In contrast, enzyme induction may accelerate drug clearance, potentially leading to therapeutic failure [8,20]. A well-known example of this is GFJ, which increases plasma levels of several CYP3A4 substrates, including simvastatin, thereby enhancing the risk of adverse effects such as myopathy and rhabdomyolysis [22].

## 4. Diet-CYP3A4 Interactions: Molecular Mechanisms

As discussed in previous sections, CYP3A4 plays a central role in the metabolism of xenobiotics and is primarily expressed in hepatic and intestinal tissues, where it exerts a major influence on drug disposition. This enzyme activity is modulated via induction and inhibition, both of which can significantly alter the pharmacokinetics and pharmacodynamics of co-administered medications, macro and micronutrients.

### 4.1. Inhibition Mechanisms

Inhibition of CYP3A4 can occur through competitive, non-competitive, or mechanism-based inhibition [23]. Competitive and non-competitive inhibitors interact reversibly with the enzyme’s active site or allosteric sites, respectively, leading to transient reductions in enzymatic activity [24,25]. Mechanism-based inhibition, by contrast, involves metabolic activation of the inhibitor to a reactive intermediate that covalently binds to the enzyme, resulting in irreversible loss of function [26,27]. Recovery from mechanism-based inhibition necessitates de novo synthesis of CYP3A4, which can take 24 to 72 h depending on tissue-specific enzyme turnover rates [28].

Among dietary inhibitors, GFJ remains a paradigmatic example due to its mechanism-based inactivation of intestinal CYP3A4 [29,30]. The primary bioactive constituents responsible for this interaction are furanocoumarins, notably bergamottin and 6′,7′-dihydroxybergamottin (DHB), both abundant in GFJ [30,31,32]. DHB is a mechanism-based inactivator, forming a metabolic intermediate complex that binds irreversibly to the enzyme, leading to its degradation and permanent loss of function, independent of the substrate used [33,34,35]. In contrast, BG exhibits a slower, reversible inhibition that is substrate-dependent, likely due to direct but transient binding to the enzyme’s active site. Within four hours of ingestion, DHB can reduce intestinal CYP3A4 content by nearly 50%, and repeated intake has been associated with a 62% decrease in enterocyte CYP3A4 levels, without altering its mRNA expression—suggesting a post-transcriptional regulatory mechanism involving increased protein degradation [7]. This effect appears isoform-selective, sparing other cytochromes such as CYP1A1 and CYP2D6 [35].

Upon ingestion, these compounds are metabolized by CYP3A4 into reactive intermediates that bind irreversibly to the enzyme’s active site, reducing its capacity to metabolize orally administered drugs with high first-pass metabolism. Studies using Caco-2 intestinal cell models have shown that DHB and related dimers cause a dose-dependent decrease in both catalytic activity and immunoreactive CYP3A4 protein levels [18].

This inhibition occurs rapidly, reaching peak effect between 30 min and 3 h after ingestion, while recovery requires 24–72 h depending on enzyme turnover, exposure level, and individual variability [36,37,38,39]. This irreversible inhibition leads to increased systemic bioavailability of affected drugs, potentially resulting in toxicity or exaggerated pharmacologic effects. Clinically significant interactions have been documented with felodipine [40,41,42], simvastatin, midazolam, cyclosporine, and many others [43]. Interestingly, other citrus fruits, such as limes, contain lower concentrations of furanocoumarins or limonin a triterpenoid that can still contribute to modest inhibition [44,45,46,47,48]. Orange juice, however, lacks significant furanocoumarin content and generally does not affect CYP3A4 [49].

Overall, GFJ remains a high-risk dietary factor for CYP3A4-mediated interactions due to its potent, irreversible inhibitory effects, extended duration of action, and impact on numerous drug substrates. Polyphenols, including flavonoids, are ubiquitous secondary metabolites found in tea, wine, fruits, vegetables, and chocolate [50].

Beyond citrus-derived furanocoumarins, polyphenols and flavonoids widely present in the human diet—such as quercetin, resveratrol, curcumin, and epigallocatechin gallate (EGCG)—have also been shown to inhibit CYP3A4 via diverse and often overlapping mechanisms [7,14,51,52,53]. These include competitive inhibition, in which the compound directly blocks the active site by mimicking the substrate structure; non-competitive inhibition, where binding occurs at allosteric sites modifying enzyme conformation; and mechanism-based inactivation, involving irreversible modification of the heme group or catalytic residues following metabolic activation. Moreover, some polyphenols may interfere with transcriptional regulation or protein stability, thereby affecting CYP3A4 levels indirectly. For example, chrysin and quercetin inhibit both constitutive and vitamin D3-induced CYP3A4 activity in intestinal Caco-2 cells (see Section 4.2 for details on VDR-mediated transcription) [54]. While these compounds are often associated with antioxidant and anti-inflammatory properties, many also exert modulatory effects on CYP3A4 [14], with both inhibitory and inductive potential depending on structure, concentration, and context [51,55]. Notably, quercetin and curcumin have been shown to prolong the half-life of CDK4/6 inhibitors, such as palbociclib and ribociclib, and decrease their intrinsic hepatic clearance, with clinically significant consequences [56].

Key polyphenolic modulators include quercetin [57] (onions, apples), epigallocatechin gallate [58] (green tea), and resveratrol [59] (grapes, red wine). These molecules interact with CYP3A4 primarily via reversible inhibition, often through competitive or mixed-type binding at the enzyme’s active site [57,60]. Structural similarity to CYP3A4 substrates facilitates this interaction. For example, epigallocatechin gallate has been shown in vitro to inhibit CYP3A4 by occupying the active site and interfering with substrate access. Similarly, quercetin acts as a mixed-type inhibitor, capable of both competitive and non-competitive interactions [52]. Resveratrol exhibits dual behavior: low doses may inhibit while high doses can induce CYP3A4 via PXR activation in hepatic cells [61].

Unlike mechanism-based inhibitors, the binding of polyphenols is reversible, and the duration of inhibition is generally short-lived, typically a few hours after post-ingestion [14,62]. Chronic consumption of polyphenol-rich diets may lead to adaptive upregulation of CYP3A4 metabolic enzymes [63]. For instance, prolonged exposure to resveratrol can activate nuclear receptors such as PXR and Aryl hydrocarbon Receptor (AhR), enhancing CYP3A4 transcription [64]. However, in vivo studies have shown conflicting results, often due to low systemic bioavailability and extensive phase II metabolism of these compounds [65]. Thus, polyphenols represent a moderate and variable risk for drug–diet interactions [66]. Although their inhibition is generally weaker and more transient than furanocoumarins, high-dose supplements or concentrated extracts could yield clinically meaningful effects, especially in sensitive populations or polypharmacy settings [66].

Structure–activity relationship studies have confirmed that specific structural features, notably hydroxylation patterns on rings A and B of the flavonoid scaffold, are essential for potent CYP3A4 inhibition. Compounds such as baicalein, scutellarein, apigenin, and amentoflavone display strong affinity for the active site, with IC50 values in the low micromolar to nanomolar range [51,52]. Interestingly, amentoflavone exhibits mixed-type inhibition, suggesting that it can simultaneously bind to the active site and exert allosteric modulation, leading to complex and unpredictable effects depending on the co-administered drug substrate.

Complementary computational docking simulations have revealed that the acetylation of hydroxyl groups, such as in modified resveratrol analogs, significantly reduces inhibitory potency by disrupting key hydrophobic interactions and hydrogen bonds with the enzyme’s catalytic pocket. These findings highlight the flexibility and complexity of the CYP3A4 binding domain, which can accommodate multiple ligands or binding orientations [53]. Furthermore, high-throughput in vitro screens involving over 40 flavonoids have confirmed a structure-dependent pattern of inhibition. Compounds like licoflavone, kushenol K, and myricetin exhibit strong, selective inhibition of CYP3A4, often with minimal cross-reactivity with other CYP isoforms, and retain their potency in primary human hepatocyte models, supporting their relevance to in vivo settings [67].

Furthermore, despite there are no direct data in humans, piperine inhibits both the drug transporter P-glycoprotein and the major drug-metabolizing enzyme CYP3A4, available data indicates that dietary piperine could affect plasma concentrations of P-glycoprotein and CYP3A4 substrates in humans, in particular if these drugs are administered orally [68].

Importantly, these inhibitory effects are modulated by a range of host-specific factors, including genetic polymorphisms in CYP3A4, individual gut microbiota profiles, and dietary context, all of which shape the extent and duration of enzyme inhibition. The co-ingestion of flavonoid-rich foods or supplements with CYP3A4 substrates may thus lead to unanticipated increases in drug plasma concentrations, posing a significant risk of toxicity, particularly for agents with narrow therapeutic windows. Consequently, understanding the molecular underpinnings of dietary CYP3A4 inhibition is crucial to anticipate and mitigate food–drug interactions in clinical practice.

### 4.2. Induction Mechanisms

Induction of CYP3A4 typically occurs via ligand-activated nuclear receptors such as the PXR and, to a lesser extent, the constitutive androstane receptor (CAR) and AhR [69]. Upon ligand binding, these receptors translocate to the nucleus, where they interact with response elements on the promoter region of the CYP3A4 gene, thereby enhancing transcription [70]. The upregulation of mRNA leads to increased protein synthesis and enzymatic activity. This process could take hours to days to manifest and persist for days after the inducing agent is removed, depending on the enzyme turnover rate [71]. It is worth mentioning that this does not consider individuals’ phenotype.

Among the most notable is St. John’s Wort (*Hypericum perforatum*), which has become a model herb for studying enzyme induction [72]. The primary active constituent, hyperforin, is a potent PXR agonist. Activation of this nuclear receptor initiates transcriptional upregulation of the CYP3A4 gene, leading to marked increases in enzyme expression [73,74]. Induction is time-dependent, requiring several days of continuous exposure to produce a significant effect. Maximal enzyme induction generally occurs within 7 to 10 days, and elevated CYP3A4 levels may persist for several days after discontinuation [75]. This enhanced enzymatic activity lowers plasma concentrations of CYP3A4 substrates, potentially resulting in therapeutic failure. Human and in vitro studies have confirmed that St. John’s Wort significantly increases the metabolism of drugs like midazolam, with stronger induction observed in females, possibly due to hormonal or epigenetic modulation of receptor activity [76].

Cruciferous vegetables, including broccoli, kale, Brussels sprouts, and cauliflower, contain a diverse array of phytochemicals with the potential to modulate xenobiotic metabolism. Notably, these vegetables are rich in glucosinolates, which upon hydrolysis yield indole-3-carbinol and isothiocyanates, such as sulforaphane. Indole-3-carbinol and its acid condensation product 3,3′-diindolylmethane can activate PXR, leading to transcriptional induction of CYP3A4 [77,78]. This induction typically starts after 3 h of intake and requires several days of sustained intake, with observed increases in enzyme expression in both hepatic and intestinal tissues. Kinetic studies suggest that enzyme induction peaks after 5–7 days of daily intake of purified compounds, and enzyme levels return to baseline within several days after withdrawals [79,80]. Interestingly, sulforaphane has also been reported to suppress both VDR- and PXR-mediated CYP3A4 induction, suggesting possible antagonistic interactions among dietary modulators [81].

Alcohol consumption represents another relevant example of dietary CYP3A4 induction. Ethanol has been shown in both in vitro and in vivo models—including CYP3A4-expressing HepG2 cells and rat liver microsomes—to increase CYP3A4 mRNA and protein levels in a dose-dependent manner. In addition to enhancing the metabolism of substrates like fentanyl, ethanol appears to stabilize the enzyme by reducing its degradation, possibly through post-translational mechanisms that modulate protein turnover [76,82].

Beyond these classical inducers, various botanical extracts and polyphenol-rich supplements have demonstrated the ability to modulate CYP3A4 expression. In primary human hepatocytes, extracts such as kava-kava, grapeseed, and silymarin induced CYP3A4 mRNA levels, although only kava-kava activated PXR-dependent luciferase expression, indicating a direct genomic mechanism. In contrast, quercetin increased CYP3A4 transcription but failed to activate PXR in reporter systems (see Section 4.1) [83].

In addition to PXR, the VDR contributes significantly to CYP3A4 transcriptional regulation. Its active ligand, 1α,25-dihydroxyvitamin D_3_ (1,25(OH)_2_D_3_), forms a heterodimer with retinoid R receptor α (RXRα) and binds to vitamin D response elements (VDREs) in the CYP3A gene promoter region, upregulating gene expression. This mechanism has been demonstrated in Caco-2 cells, hepatocytes cultured in 2D and 3D, and in transgenic mice, with the most pronounced effects occurring in the intestine [81,83,84].

Moreover, essential oils (EOs) from common culinary herbs such as oregano, rosemary, and thyme have been shown to induce CYP3A4 expression via PXR activation in both intestinal and hepatic human models. The absence of this effect in PXR-knockout cells confirms that EO-mediated CYP3A4 induction is strictly PXR-dependent, indicating that everyday food seasonings may influence drug metabolism through sustained receptor activation [85].

Experimental data from animal models have also highlighted the role of endogenous dietary components such as bile acids and sterols. In humanized CYP3A4 transgenic mice, diets enriched in cholesterol and cholic acid upregulated hepatic CYP3A4 expression and accelerated triazolam clearance. These findings suggest a modulatory effect via PXR or FXR activation, particularly relevant in metabolic disease contexts [86].

Among functional foods, *Coleus forskohlii* has attracted significant attention for its strong CYP3A4-inducing capacity. Its active compound, forskolin, exhibited the highest transcriptional activation in hepatocyte-derived luciferase assays. In humanized PXR–CYP3A4 mice, forskolin increased hepatic enzyme expression nearly fourfold and enhanced the formation of hepatotoxic metabolites of acetaminophen, highlighting a clinically relevant supplement–drug interaction with potential hepatotoxic implications [87].

Finally, preclinical studies using rat liver microsomes have demonstrated that extracts of hibiscus and *Tradescantia zebrina* upregulate CYP3A4 activity, as evidenced by enhanced aminopyrine metabolism. In contrast, Chinese cabbage juice was found to inhibit the enzyme, reinforcing the bidirectional nature of phytochemical modulation and underscoring the importance of evaluating food–drug interactions on a compound-specific basis [88].

In summary, dietary and botanical induction of CYP3A4 involves a complex network of nuclear receptor activation, mRNA transcription, post-transcriptional stabilization, and protein protection mechanisms. Given that CYP3A4 is responsible for the metabolism of over 50% of clinically used drugs, its upregulation can lead to subtherapeutic plasma concentrations, reduced efficacy, or shortened drug half-lives. Therefore, clinical awareness of CYP3A4 inducers, especially from herbal supplements and functional foods, is essential to prevent therapeutic failure in polymedicated patients.

### 4.3. Epigenetic and Microbiota Modulation

#### 4.3.1. Epigenetic Regulation of CYP3A4

As mentioned earlier, CYP3A4 is the most abundant and pharmacologically relevant cytochrome P450 enzyme in humans, regulated by genetic, epigenetic, and environmental inputs [84,89]. In addition to classical nuclear receptor pathways, dietary components can influence its transcription through epigenetic mechanisms such as DNA methylation, histone modifications, and microRNA (miRNA)-mediated regulation [14,84,89].

Specifically, bioactive compounds like polyphenols, vitamins, and methyl-donor nutrients have been shown to act at these regulatory layers. Some polyphenols, such as quercetin, can modulate CYP3A4 expression through epigenetic mechanisms (see Section 4.1), while vitamins like 1α,25-dihydroxyvitamin D_3_ act via VDR-mediated transcription (see Section 4.2). with PXR and CAR [90,91]. This coordinated action likely involves chromatin remodeling and cross-talk among nuclear receptors, thereby integrating dietary, hormonal, and xenobiotic signals [91].

Importantly, such epigenetic regulation may contribute to the marked interindividual variability in drug metabolism, as DNA methylation patterns, histone modifications, and non-coding RNAs dynamically modulate CYP3A4 expression in a tissue- and context-specific manner [92,93]. In this context, curcumin serves as a notable example: in aflatoxin B1-exposed broiler models, it suppresses CYP3A4 expression and restores methylation balance by upregulating DNA methyltransferases (DNMT1, DNMT3a, DNMT3b) [94]. Similarly, nutrients participating in one-carbon metabolism—such as folate, vitamins B6/B12, and choline—provide methyl groups or cofactors for S-adenosylmethionine (SAM) synthesis, thereby indirectly influencing DNA methylation and transcriptional activity [91].

Beyond DNA methylation, histone modifications also play a pivotal role. For example, butyrate—a short-chain fatty acid produced by gut microbiota during fiber fermentation—acts as a histone deacetylase (HDAC) inhibitor, increasing histone acetylation and thus promoting a more open chromatin structure that can enhance xenobiotic metabolism, potentially including CYP3A4 [95,96]. Similarly, dietary polyphenols such as resveratrol and quercetin have demonstrated the capacity to inhibit HDAC activity, contributing to a chromatin state permissive for gene expression [97].

Finally, miRNAs add yet another regulatory layer by binding to the 3′ untranslated regions (3′-UTRs) of target mRNAs, leading to degradation or translational repression. Evidence indicates that dietary polyphenols, vitamins, and methyl donors can modulate miRNA transcription, processing, and maturation [98,99,100,101]. While a direct link between diet-regulated miRNAs and CYP3A4 remains to be fully elucidated, several studies suggest that compounds such as resveratrol, quercetin, and vitamin D can alter miRNA profiles in hepatic and intestinal models, potentially contributing to the epigenetic regulation of drug-metabolizing enzymes. This indirect regulatory axis adds complexity to the nutriepigenomic modulation of pharmacokinetics and detoxification pathways.

#### 4.3.2. Microbiota-Derived Epigenetic Modulators

The gut microbiota, through its metabolic activity and structural components, exerts a significant influence on host gene expression, including the regulation of xenobiotic-metabolizing enzymes such as CYP3A4 [83,102]. Rather than acting through direct genetic regulation, this influence is mediated via microbial metabolites and signals that integrate into host epigenetic pathways.

One key example is the short-chain fatty acid (SCFA) butyrate, produced by taxa such as *Faecalibacterium prausnitzii* and *Roseburia* spp. As described in Section 4.3.1., butyrate functions as an HDAC inhibitor; in the gut-liver axis, this activity may contribute to the transcriptional modulation of CYP3A4 alongside its established roles in maintaining intestinal barrier integrity and immune homeostasis [83].

In addition to SCFAs, other microbial metabolites such as indoles (from tryptophan metabolism), phenolic acids, and secondary bile acids have also been implicated in the epigenetic modulation of host gene expression. These compounds can interact with nuclear receptors such as the PXR and the AhR. For instance, the microbial metabolite indole-3-propionic acid (IPA) has been shown to activate AhR, leading to downstream transcriptional effects that may contribute—directly or indirectly—to epigenetic remodeling and CYP3A4 expression [14].

The gut microbiota also influences host DNA methylation through its role in methyl-donor metabolism. Bacterial production of folate, choline, and vitamin B12 contributes to the host’s one-carbon cycle, affecting intracellular levels of S-adenosylmethionine (SAM), the universal methyl donor for DNA and histone methylation reactions. Perturbations in microbial composition—such as those induced by diet, antibiotics, or disease—can alter these metabolic fluxes, ultimately impacting gene-specific methylation profiles, including those of CYP genes [102].

Additionally, microbial activity can influence host miRNA networks. Certain strains and metabolites modulate miRNA transcription or processing, including miR-27b, which targets PXR mRNA and thus indirectly regulates CYP3A4 expression [90]. Likewise, bacterial components such as lipopolysaccharides (LPS) and extracellular vesicles have been reported to modulate host miRNA responses, influencing nuclear receptor signaling pathways including AhR and PXR [100].

The interplay between microbiota and epigenetic regulation is further supported by studies demonstrating interindividual variability in CYP3A4 expression, which may be partially attributed to differences in microbial composition and metabolite profiles. These findings highlight the potential of the gut microbiota to shape drug metabolism through nutrient-sensitive, epigenetically mediated pathways, although more mechanistic studies are needed to define these interactions in humans [103].

## 5. Dietary and Pharmacological Modulators of CYP3A4: Clinical Relevance for Statins and Antidiabetic Drugs

### 5.1. Lipid-Lowering Drugs and CYP3A4

Lipid-Lowering Drugs and CYP3A4 Lipid-lowering drugs, particularly statins, are widely prescribed to reduce low-density lipoprotein cholesterol (LDL-C) and prevent cardiovascular diseases. Statins inhibit HMG-CoA reductase—the rate-limiting enzyme in hepatic cholesterol biosynthesis—thereby lowering intracellular cholesterol levels and promoting LDL receptor expression [104].

Most statins are substrates of CYP3A4, making them vulnerable to pharmacokinetic interactions with substances that inhibit or induce this enzyme. Inhibition of CYP3A4 can elevate plasma statin concentrations, heightening the risk of adverse effects such as myopathy and rhabdomyolysis, particularly for statins with low oral bioavailability. Conversely, enzyme induction may reduce drug efficacy. Extensive first-pass metabolism in the liver and intestine contributes to this low bioavailability. Statins are mainly eliminated via biliary excretion, with minimal renal involvement [20,105].

#### 5.1.1. Simvastatin

Simvastatin is a lipophilic statin administered as an inactive lactone prodrug. Once absorbed, it is hydrolyzed in the liver to simvastatin acid, its active β-hydroxyacid form, which inhibits HMG-CoA reductase and enhances LDL clearance [106].

CYP3A4 plays a predominant role in the metabolism of simvastatin, acting in both hepatic and intestinal tissues. It catalyzes oxidative conversion of the lactone ring into inactive metabolites, limiting the bioavailability of the drug to less than 5% [106,107]. As a result, simvastatin is highly susceptible to interactions with strong CYP3A4 inhibitors, such as ketoconazole, erythromycin, and ritonavir, which markedly elevate plasma concentrations and increase the risk of muscle-related adverse events [106,107].

Following administration, simvastatin acid reaches peak plasma concentrations within 1.3 to 2.4 h. The compound exhibits high plasma protein binding (>95%) and is mainly eliminated in bile, with under 13% excreted in urine. Its terminal half-life is approximately 1.9 h [106]. It does not notably inhibit or induce other CYP isoforms, minimizing the likelihood of further pharmacokinetic interactions [106].

Efforts to enhance simvastatin’s bioavailability have focused on modified-release formulations that deliver the drug to distal intestinal regions with reduced CYP3A4 expression. One study demonstrated a threefold increase in systemic exposure when simvastatin was released in such areas [107].

#### 5.1.2. Atorvastatin

Atorvastatin is a lipophilic statin administered in its active hydroxyacid form. It competitively inhibits HMG-CoA reductase, reducing intracellular cholesterol levels and stimulating LDL receptor expression, which facilitates the clearance of LDL cholesterol from circulation [108].

This statin undergoes extensive metabolism via cytochrome P450 3A4 (CYP3A4), both in the liver and the intestinal mucosa. CYP3A4 converts atorvastatin into active ortho- and para-hydroxylated metabolites, as well as into inactive lactone derivatives. The lactone form has a higher affinity for CYP3A4. This metabolic profile makes atorvastatin vulnerable to interactions with CYP3A4 inhibitors, such as itraconazole, ritonavir, and GFJ, potentially raising systemic drug levels and the risk of adverse effects like myopathy or rhabdomyolysis [109].

First-pass metabolism by enterocytes and hepatocytes reduces its oral bioavailability to approximately 14% [110]. Although CYP3A4 is the major metabolic pathway, atorvastatin is also subject to glucuronidation by UGT1A1 and UGT1A3. Notably, it does not significantly inhibit or induce other CYP enzymes, which minimizes the potential for widespread pharmacokinetic interactions [108].

Atorvastatin exhibits high plasma protein binding (95–98%) [111] and reaches peak concentrations within 1–2 h after administration [112]. The drug is mainly excreted via the biliary route, with renal clearance contributing less than 1% to its elimination. Its terminal half-life is about 7 h, although pharmacodynamic effects last longer due to prolonged HMG-CoA reductase inhibition [108].

Advanced drug delivery systems, such as dry emulsions, co-amorphous formulations, and microencapsulation, have been developed to enhance oral absorption by improving solubility and reducing the impact of intestinal metabolism [113].

#### 5.1.3. Lovastatin

Lovastatin is a lipophilic statin and a lactone prodrug. After oral administration, it is hydrolyzed in the liver to form lovastatin acid, the active β-hydroxyacid that inhibits HMG-CoA reductase and promotes LDL receptor expression, increasing LDL clearance from the plasma [114].

CYP3A4 is primarily responsible for lovastatin’s oxidative metabolism, especially in the intestinal epithelium. This extensive first-pass effect contributes to its low systemic bioavailability, which is less than 5% [115]. Though minor contributions from CYP2C9 and CYP2D6 have been observed in vitro, their role in clinical settings is considered negligible. Due to its reliance on CYP3A4, co-administration with potent inhibitors such as erythromycin, ketoconazole, or GFJ may substantially increase plasma concentrations, elevating the risk of muscle-related adverse events, including rhabdomyolysis [20,114].

Pharmacokinetically, lovastatin is characterized by a high degree of plasma protein binding (>95%). Peak plasma concentrations of lovastatin acid are reached between 2 and 4 h after dosing. The compound is primarily excreted through the biliary route, with renal elimination accounting for less than 10% of its clearance. Its terminal half-life is approximately 3 h; however, its lipid-lowering effects endure longer due to persistent inhibition of its enzymatic target [114,115].

Lovastatin does not significantly modulate the activity of other CYP isoforms, which limits the potential for broad pharmacokinetic interactions. To address its low oral bioavailability and improve clinical performance, novel formulation strategies such as solid lipid nanoparticles and self-emulsifying drug delivery systems have been explored. These technologies aim to enhance solubility and reduce the extent of intestinal metabolism, thereby improving systemic exposure [116].

#### 5.1.4. Cerivastatin

Cerivastatin is a synthetic and enantiomerically pure statin administered in its active acid form. It possesses exceptionally high potency as an HMG-CoA reductase inhibitor, with a Ki of approximately 1.3 nM, allowing therapeutic efficacy at microgram-level doses [117]. After oral intake, cerivastatin is rapidly and completely absorbed, achieving peak plasma concentrations within 2 to 3 h. It displays linear pharmacokinetics, with dose-proportional increases in maximum plasma concentration (Cmax) and area under the concentration–time curve (AUC). More than 99% of the circulating drug is bound to plasma proteins. Its moderate volume of distribution reflects limited tissue penetration but a preferential accumulation in hepatic tissue, the primary site of action [118].

Cerivastatin is eliminated exclusively through metabolic pathways, with no excretion of the parent drug in its unchanged form. Two main oxidative biotransformation pathways are involved: (1) demethylation of the benzylic methyl ether group to form metabolite M-1, catalyzed by CYP2C8 and CYP3A4, and (2) stereoselective hydroxylation of the 6-isopropyl moiety to produce metabolite M-23, primarily catalyzed by CYP2C8 [118,119]. Both metabolites maintain inhibitory activity against HMG-CoA reductase and are excreted through fecal (~70%) and renal (~30%) pathways [117].

The existence of dual metabolic routes offers some protective redundancy against CYP-mediated inhibition. If one pathway is blocked, the alternative route may partially compensate. Nevertheless, co-administration with strong CYP2C8 inhibitors such as gemfibrozil has been shown to markedly elevate cerivastatin and metabolite levels. This pharmacokinetic interaction significantly increases the risk of serious adverse outcomes, including rhabdomyolysis, which ultimately led to the drug’s withdrawal from the market in several countries [120,121].

### 5.2. Antidiabetic Drugs and CYP3A4

The relevance of CYP3A4 in the pharmacokinetics of antidiabetic agents lies in its susceptibility to both inhibition and induction, processes that can dramatically alter the plasma concentrations and therapeutic efficacy of these drugs [122,123]. In clinical practice, this is especially relevant because patients with type 2 diabetes mellitus often receive multiple medications for comorbidities such as dyslipidemia, hypertension, and cardiovascular disease [124], increasing the risk of drug–drug interactions [125] and interindividual variability due to genetic polymorphisms affecting CYP3A4 expression or function.

Several oral antidiabetic agents are known to be substrates of CYP3A4. These include troglitazone [5,126], linagliptin [127], and pioglitazone [128,129], among others. In some cases, CYP3A4 is the primary metabolic route, while in others, it acts in concert with other isoenzymes such as CYP2C8 [130] or CYP2C9 [3]. For instance, pioglitazone is primarily metabolized by CYP2C8, but CYP3A4 also plays a secondary role [131]. Similarly, repaglinide is biotransformed mainly by CYP2C8 and CYP3A4 [130], and saxagliptin is extensively metabolized by CYP3A4/5 [132].

The modulation of CYP3A4 by co-administered drugs or dietary constituents may lead to clinically relevant pharmacokinetic alterations [14,15,133]. Potent inhibitors such as ketoconazole, clarithromycin, or cyclosporine can increase drug exposure, raising the risk of adverse effects like hypoglycemia [134,135]. Moreover, certain foods and herbal supplements can also inhibit or induce CYP3A4 activity, complicating the management of diabetes therapy [136,137].

Understanding the role of CYP3A4 in the metabolism of antidiabetic drugs is essential for preventing drug interactions and ensuring therapeutic efficacy. This is especially critical in patients with renal or hepatic impairment, elderly populations, or those with polypharmacy [138,139,140]. The following subsections will explore the metabolic pathways and interaction profiles of the main antidiabetic agents influenced by CYP3A4, including mechanistic insights and clinical implications.

#### 5.2.1. Repaglinide

Repaglinide is an oral insulin secretagogue of the meglitinide class, used primarily for the management of postprandial hyperglycemia in patients with type 2 diabetes mellitus. It is particularly indicated in individuals with irregular meal patterns due to its rapid onset and short duration of action [141].

Mechanistically, repaglinide stimulates insulin secretion by binding to and inhibiting ATP-sensitive potassium channels on the pancreatic β-cell membrane [142]. This results in membrane depolarization and opening of voltage-gated calcium channels, leading to an influx of calcium and subsequent exocytosis of insulin-containing granules [143]. Unlike sulfonylureas, repaglinide exhibits rapid dissociation from the sulfonylurea receptor 1 (SUR1) subunit of the K-ATP channel, which may reduce the risk of prolonged hypoglycemia and allow for a more flexible dosing regimen based on meals [144].

Pharmacokinetically, repaglinide is rapidly absorbed, reaching peak plasma concentrations within one hour, and has a plasma half-life of approximately 1–1.5 h [144]. It undergoes almost complete hepatic metabolism, with negligible renal excretion of the unchanged drug [145]. The biotransformation of repaglinide is primarily mediated by CYP2C8 and CYP3A4, and to a lesser extent, by uridine diphosphate-glucuronosyltransferases [130,146].

CYP2C8 is the dominant enzyme responsible for the formation of the active metabolite M4, while CYP3A4 predominantly generates M1 and other minor metabolites [130]. This dual metabolic pathway renders repaglinide susceptible to interactions with drugs that inhibit or induce either enzyme. In particular, the glucuronide metabolite of clopidogrel is a strong CYP2C8 inhibitor and significantly increases repaglinide plasma concentrations, elevating the risk of hypoglycemia [147]. Similarly, gemfibrozil, a lipid-lowering agent, inhibits both CYP2C8 and the hepatic uptake transporter OATP1B1, leading to an 8-fold increase in repaglinide exposure [148]. Given this dual enzymatic pathway, the potential for pharmacokinetic interactions with CYP2C8 and CYP3A4 inhibitors is clinically significant. Notably, concomitant use of repaglinide and gemfibrozil is contraindicated due to a markedly increased risk of hypoglycemia.

On the other hand, CYP3A4 inducers such as rifampicin can significantly reduce repaglinide plasma levels. In a controlled study, administration of rifampicin 24 h before repaglinide significantly reduced its AUC by approximately 80%, which may lead to a clinically relevant decrease in its glucose-lowering efficacy [149]. Moreover, inhibitors of CYP3A4 such as itraconazole or clarithromycin can modestly increase repaglinide exposure [150], although not to the same extent as CYP2C8 inhibitors [151].

Additionally, cyclosporine, a known inhibitor of both CYP3A4 and OATP1B1, markedly increased repaglinide plasma concentrations in healthy volunteers (by 2.4-fold in AUC). This pharmacokinetic interaction was significantly modulated by SLCO1B1 polymorphisms (e.g., rs4149056), with a reduced effect observed in individuals carrying the 521TC genotype, compared to those with the 521TT reference genotype [151]. These findings highlight the importance of considering both metabolic enzymes and transporters when predicting pharmacokinetic interactions.

Due to its narrow therapeutic window and risk of severe hypoglycemia, especially in elderly patients or those with renal impairment, careful consideration of repaglinide’s metabolic pathways is essential in clinical practice.

#### 5.2.2. Nateglinide

Nateglinide is another member of the meglitinide class of oral antidiabetic agents, sharing structural and mechanistic similarities with repaglinide [152]. As previously described for this drug class, nateglinide promotes glucose-dependent insulin secretion by targeting ATP-sensitive potassium channels on pancreatic β-cells via interaction with the SUR1 subunit [144]. However, compared to repaglinide, nateglinide has an even faster onset and shorter duration of action, leading to a more pronounced early-phase insulin release, which is particularly effective in attenuating postprandial hyperglycemia [153].

Pharmacokinetically, nateglinide is rapidly absorbed after oral administration, reaching peak plasma concentrations within 0.5 to 1 h [154]. Its half-life is approximately 1.5 h, and its glucose-lowering effect is brief, making it suitable for administration immediately before meals [154]. The drug undergoes extensive hepatic metabolism, primarily through CYP2C9 and, to a lesser extent, CYP3A4, yielding hydroxylated and conjugated metabolites with negligible pharmacological activity [155].

In contrast to repaglinide, which is also influenced by CYP2C8 and hepatic uptake transporters such as OATP1B1, nateglinide exhibits a more limited interaction profile [156]. Nevertheless, coadministration with strong CYP inducers like rifampicin has been shown to reduce its bioavailability and compromise its efficacy [4]. Rifampicin significantly accelerates nateglinide clearance, likely through simultaneous induction of both CYP3A4 and CYP2C9 [157]. In contrast, coadministration with CYP inhibitors such as clarithromycin has shown minimal effect on nateglinide exposure, whereas potent inhibitors like fluconazole and miconazole significantly increase its plasma levels due to strong inhibition of CYP2C9 and CYP3A4 [158].

However, in patients with reduced CYP2C9 activity, due to polymorphisms (e.g., CYP2C9 *2/*2 or *3/*3 genotypes) or pharmacologic inhibition [3], the contribution of CYP3A4 becomes more significant. In these cases, co-administration with CYP3A4 inhibitors could lead to accumulation of nateglinide [158].

Due to its rapid onset and short duration of action, along with glucose-dependent insulinotropic properties, nateglinide is considered a suitable option for patients in the early stages of type 2 diabetes or those with irregular mealtime patterns, offering flexibility and a reduced risk of prolonged hypoglycemia [159]. Nonetheless, its dual metabolic dependence on CYP2C9 and CYP3A4 requires consideration in polypharmacy scenarios, especially when inhibitors or inducers of both enzymes are present.

#### 5.2.3. Saxagliptin

Saxagliptin is an oral antidiabetic agent belonging to the class of dipeptidyl peptidase-4 (DPP-4) inhibitors [160]. It is indicated for the treatment of type 2 diabetes mellitus, primarily as an adjunct to diet and exercise to improve glycemic control either in monotherapy or in combination with other antidiabetic drugs, including metformin and sulfonylureas [160].

The therapeutic mechanism of saxagliptin involves the inhibition of the enzyme DPP-4, a serine protease that rapidly inactivates incretin hormones such as glucagon-like peptide-1 (GLP-1) and glucose-dependent insulinotropic peptide (GIP) [160]. These incretins play a crucial role in maintaining glucose homeostasis by stimulating insulin secretion from pancreatic β-cells and suppressing glucagon release from α-cells in a glucose-dependent manner. By prolonging the activity of endogenous incretins, saxagliptin enhances postprandial insulin response and reduces hepatic glucose production, thereby improving both fasting and postprandial plasma glucose levels [160].

Pharmacokinetically, saxagliptin is rapidly absorbed after oral administration. Peak plasma concentrations are reached at approximately 4 h for saxagliptin and 2 h for its active metabolite M2 [160]. It undergoes extensive hepatic metabolism, primarily mediated by cytochrome P450 3A4 and CYP3A5, forming the principal active metabolite 5-hydroxysaxagliptin, which circulates in plasma and contributes significantly to the drug’s overall DPP-4 inhibitory effect [161,162].

In a recent in vitro study, Liu et al. [163] confirmed that CYP3A4 plays a central role in the oxidative metabolism of saxagliptin. By evaluating the catalytic efficiency of 27 CYP3A4 variants, the authors observed substantial differences in enzymatic activity, with many variants showing a 1.9–7% reduction in intrinsic clearance compared to the wild type [163]. These polymorphisms may lead to slower metabolism, higher parent drug concentrations, and a potential increased risk of adverse effects such as hypoglycemia, especially in combination regimens [163].

Clinically, saxagliptin is susceptible to pharmacokinetic interactions with potent CYP3A4 inhibitors and inducers [164,165]. In the presence of strong CYP3A4 inhibitors, saxagliptin exposure may increase, which could necessitate careful monitoring or dose adjustments [165]. Conversely, enzyme induction may reduce saxagliptin levels, which could potentially diminish its therapeutic effect [164].

Given its reliance on CYP3A4-mediated metabolism, saxagliptin may also be affected by dietary constituents known to inhibit this enzyme, such as GFJ or certain herbal supplements. Clinical pharmacology data show that moderate CYP3A4 inhibitors like GFJ can increase saxagliptin exposure, though current guidance does not mandate dose adjustments [166,167]. However, clinical studies evaluating such interactions remain limited, and current guidelines do not mandate routine avoidance of these foods in patients receiving saxagliptin.

#### 5.2.4. Canagliflozin

In addition to DPP-4 inhibitors, other antidiabetic classes, such as SGLT2 inhibitors, may also be subject to CYP3A4-mediated metabolic modulation, albeit to a lesser extent. Canagliflozin is a sodium–glucose cotransporter 2 (SGLT2) inhibitor that has been approved for the management of T2DM and is increasingly recognized for its pleiotropic benefits. Its mechanism of action is independent of insulin secretion and involves inhibiting glucose reabsorption in the proximal renal tubules, thereby increasing glucosuria and improving glycemic control [168]. Notably, it reduces fasting and postprandial glucose without increasing the risk of hypoglycemia [169].

Canagliflozin has demonstrated cardiovascular and renal protective effects, which are thought to be mediated not only by glucose-lowering but also by additional mechanisms such as reductions in systolic blood pressure (−3.93 mmHg), body weight (−1.6 kg), and albuminuria [170]. Neal et al. also reported improved lipid profiles and a decreased need for other glucose-lowering agents. Moreover, Polidori et al. [171] observed significant improvements in beta-cell glucose sensitivity and insulin secretion rates in patients treated with canagliflozin, suggesting a recovery of pancreatic function likely due to reduced glucotoxicity.

Once orally administered, canagliflozin undergoes hepatic metabolism predominantly through glucuronidation (via UGT1A9 and UGT2B4) [172] and, to a lesser extent, oxidative metabolism mediated by CYP3A4 [173]. While CYP3A4 is not the major metabolic route, it may play a clinically significant role in scenarios involving strong CYP3A4 inducers (e.g., rifampicin), but not with inhibitors.

This is supported by clinical findings showing that co-administration with rifampicin reduced canagliflozin plasma concentrations by approximately 51% in overall exposure and by 28% in peak concentration, potentially compromising its glucose-lowering efficacy. Therefore, dose adjustment or closer monitoring may be warranted when used concomitantly with strong CYP3A4 inducers [173].

In terms of elimination, canagliflozin exhibits a dual excretion pathway: approximately 60% of the administered dose is excreted in feces, primarily as unchanged drug (41.5%) due to incomplete absorption and biliary secretion of glucuronidated metabolites (M7, M5), which undergo intestinal hydrolysis back to the parent compound. Renal excretion accounts for 33% of the dose, predominantly as inactive O-glucuronide metabolites (M5: 13.3%, M7: 17.2%), with less than 1% excreted as intact canagliflozin. The drug’s low renal clearance (0.049–0.13 L/h) reflects its high plasma protein binding (98–99%) and minimal active tubular secretion [174].

#### 5.2.5. Pioglitazone

Pioglitazone is an oral antidiabetic drug belonging to the thiazolidinedione (TZD) class. It is widely used as an insulin sensitizer in the treatment of type 2 diabetes mellitus, particularly in patients with insulin resistance and features of metabolic syndrome. Its primary mechanism of action is mediated by activation of the nuclear transcription factor peroxisome proliferator-activated receptor gamma (PPARγ), which is highly expressed in adipose tissue and, to a lesser extent, in skeletal muscle, liver, and the vasculature [126].

Upon activation, PPARγ forms a heterodimer with the RXR and binds to specific peroxisome proliferator response elements (PPREs) in the promoter regions of target genes. This leads to modulation of gene transcription involved in glucose and lipid metabolism, adipogenesis, insulin signaling, and anti-inflammatory pathways. As a result, pioglitazone enhances insulin sensitivity in peripheral tissues by promoting glucose uptake (primarily via increased GLUT4 translocation in skeletal muscle and adipocytes), suppresses hepatic gluconeogenesis, and improves lipid profiles by reducing circulating free fatty acids and triglycerides [126].

In terms of metabolism, pioglitazone is primarily biotransformed in the liver by cytochrome P450 enzymes. Although CYP2C8 is the main isoenzyme responsible for its clearance, CYP3A4 contributes to approximately 37% of its metabolism in vitro [131]. This secondary role becomes more clinically relevant in the presence of enzyme modulators. For example, CYP3A4 inhibitors such as ketoconazole or clarithromycin may modestly increase pioglitazone plasma levels, whereas inducers like rifampicin can reduce its systemic exposure, potentially diminishing its therapeutic efficacy [131,175]. The role of CYP3A4 becomes even more important in patients with genetic polymorphisms or drug–drug interactions affecting CYP2C8, where CYP3A4 may partially compensate [131,176]. Understanding these interactions is critical for optimizing dosing and avoiding adverse effects [175].

Beyond glycemic control, pioglitazone has been shown to exert pleiotropic effects such as improvement in endothelial function, reduction in inflammatory markers (e.g., CRP, TNF-α, IL-6) [177], and even anti-atherogenic properties. However, its use is sometimes limited by side effects such as fluid retention, weight gain, and concerns about cardiovascular safety and bone health.

For clarity and to facilitate comparison, a concise summary of the pharmacokinetic characteristics and CYP3A4-related interactions of these agents is presented in Table 1.

## 6. Evidence from Clinical and Preclinical Studies

### 6.1. Citrus Compounds and Furanocoumarins

As previously detailed, GFJ is a well-established dietary inhibitor of intestinal CYP3A4. The following preclinical and clinical studies illustrate the impact of this interaction on various pharmacological agents, including statins and antidiabetic drugs.

In a preclinical study in hyperlipidaemic Wistar rats, the co-administration of atorvastatin (2.5 mg/kg) with bergamottin led to a notable increase in the Cmax of atorvastatin (from 48 ± 5 to 89 ± 7 ng/mL) and a threefold elevation in AUC_0–∞_ (from 176 ± 27 to 552 ± 131 h·μg/L). Additionally, the elimination half-life (t_1/2_) of atorvastatin was prolonged, indicating a slower systemic clearance. These pharmacokinetic alterations translated into enhanced pharmacodynamic effects, with significant reductions in total cholesterol (−14%), LDL-cholesterol (−20%) and triglycerides (−12%), alongside an increase in HDL-cholesterol. The findings support the notion that bergamottin can potentiate the therapeutic efficacy of atorvastatin, though with a potential risk of increased toxicity due to drug accumulation [178].

Human studies have confirmed these effects. A crossover trial comparing grapefruit and pomegranate juice found that GFJ significantly increased the Cmax and AUCinf of simvastatin (15.6-fold and 9.1-fold, respectively), whereas pomegranate juice had no effect, suggesting minimal CYP3A4 inhibition by the latter [179].

The inhibitory action of bergamottin on simvastatin metabolism has also been characterized in vitro. In rat liver microsomes, the inhibition was more pronounced following pre-incubation, especially with NADPH, indicating potential mechanism-based inhibition. The Ki values ranged from 174 ± 36 μM during co-incubation to as low as 4 ± 2 μM during NADPH-dependent pre-incubation. In contrast, in human liver microsomes, bergamottin exhibited consistent inhibitory activity across conditions, with Ki values between 22 and 34 μM. These results reflect both species differences and the importance of CYP-mediated metabolite formation in determining inhibitor potency [180].

Furthermore, flavonoids like naringenin, also present in GFJ, demonstrated comparable inhibitory effects on simvastatin metabolism. While both bergamottin and naringenin showed mixed-type inhibition in human microsomes, naringenin had a lower Ki (29 ± 11 μM) than bergamottin (34 ± 5 μM), suggesting potent inhibition from both compound classes. Interestingly, in rat liver microsomes, the inhibitory potency of naringenin surpassed that of bergamottin, again underscoring interspecies variability [180].

In primary rat hepatocytes, the intrinsic clearance of simvastatin decreased from 26.2 μL/min/10^6^ cells to 4.15 μL/min/10^6^ cells in the presence of 50 μM naringenin. These in vitro findings support the relevance of using hepatocyte-based models when extrapolating metabolic interactions to in vivo scenarios, given the greater inhibitory effect seen in cellular systems compared to microsomes [181].

A crossover study explored the clinical impact of GFJ on statin pharmacokinetics. It demonstrated a 2.5-fold increase in the AUC_0–72_ of atorvastatin acid and a 3.3-fold increase in that of atorvastatin lactone, with significant increases in tmax and t_1/2_. Notably, GFJ reduced the levels of 2-hydroxyatorvastatin acid and lactone, which are metabolites formed via CYP3A4. These alterations led to an overall increase in active and total HMG-CoA reductase inhibitory activity (1.3-fold and 1.5-fold, respectively), likely due to reduced intestinal metabolism [182].

This differential effect across statins was further clarified in a study comparing atorvastatin and pitavastatin. GFJ increased the AUC of atorvastatin acid by 83%, while pitavastatin acid levels rose by only 13%, suggesting that pitavastatin is minimally affected by CYP3A4-mediated metabolism [183]. Similarly, pravastatin showed negligible pharmacokinetic changes with GFJ intake, reinforcing its status as a CYP3A4-independent statin [184].

Importantly, long-term administration of GFJ (300 mL/day for 90 days) in patients on chronic atorvastatin treatment resulted in only modest increases in serum atorvastatin concentrations (19–26%), with no significant changes in lipid profile or markers of liver or muscle toxicity. This suggests that moderate, chronic GFJ consumption does not necessarily necessitate atorvastatin dose reduction [185].

Further studies have delineated the time course of CYP3A4 inhibition. When simvastatin was administered with high-dose GFJ, the AUC of simvastatin increased by 13.5-fold. However, this effect diminished over time; simvastatin given 24 h later showed only a 2.1-fold increase, and after 3–7 days, no significant differences were observed compared to control, indicating a reversible and transient inhibition of CYP3A4 [36].

In the context of transporter-mediated interactions, GFJ also inhibits intestinal uptake transporters such as OATP2B1 and OATP1A2. A knockout mouse model for OATP2B1 revealed that fexofenadine absorption decreased significantly following GFJ ingestion, but not for rosuvastatin, demonstrating substrate specificity [186]. Similarly, in healthy volunteers, GFJ—but not atorvastatin—reduced the exposure of several small-dose OATP2B1 substrates (e.g., sulfasalazine, glibenclamide, celiprolol), confirming its role as an inhibitor of intestinal influx transporters [187].

Aliskiren, an antihypertensive renin inhibitor, is another example where GFJ reduced systemic exposure by 38–61%. The mechanism was traced to OATP1A2 inhibition by naringin, a major flavonoid in GFJ, rather than OATP2B1, as shown in HEK293 cell assays [188]. The variable effects of GFJ on drug efflux and absorption also extend to P-glycoprotein (P-gp). In Wistar rats, chronic GFJ administration increased intestinal P-gp expression while reducing OATP activity, resulting in decreased systemic availability of diltiazem and affecting its gut permeability [189].

Lastly, while lovastatin is also metabolized via CYP3A4, the interaction magnitude appears more moderate. In healthy subjects, regular-strength GFJ approximately doubled the AUC and Cmax of lovastatin, with a 1.6-fold increase in lovastatin acid. However, the increase in HMG-CoA reductase inhibitory activity was only around 30–40%, suggesting that GFJ’s effect on this statin may be clinically less significant than with simvastatin or atorvastatin [190].

A comparable interaction pattern has also been observed with certain antidiabetic agents, particularly those with a high dependence on intestinal CYP3A4 for metabolic clearance. One of the clearest examples of CYP3A4-mediated interaction is found in repaglinide, a short-acting meglitinide analog used to control postprandial glycemia. Repaglinide is primarily metabolized by CYP3A4, and its intestinal metabolism appears particularly susceptible to inhibition by GFJ. In a randomized, crossover clinical trial, a single dose of GFJ (300 mL) increased the AUC_0–∞_ of repaglinide by 13% and its Cmax by 5%, without significantly altering the elimination half-life. The effect was more evident at a subtherapeutic dose (0.25 mg) than at the clinical dose of 2 mg, highlighting the relevance of intestinal CYP3A4 inhibition in early absorption phases [191].

A similar interaction has been observed with glibenclamide (glyburide), although its metabolism primarily involves CYP2C9 rather than CYP3A4. In a study comparing the effects of clarithromycin and GFJ, only clarithromycin—a known P-glycoprotein inhibitor—significantly increased the AUC of glibenclamide by 35%. In contrast, GFJ had no significant effect on glibenclamide plasma concentrations, supporting the conclusion that glibenclamide is not substantially metabolized by CYP3A4, and that GFJ does not interfere with its pharmacokinetics through this pathway [192].

Interestingly, the effect of GFJ on metformin, a drug that is not metabolized by CYP enzymes but whose distribution depends on organic cation transporters, reveals indirect but clinically important interactions. In a rat model, GFJ significantly increased hepatic concentrations of metformin (397 ± 19 µg/g vs. 280 ± 15 µg/g in controls), while plasma levels remained unchanged. Although not mediated by CYP3A4, this accumulation was associated with a marked increase in blood lactic acid levels, especially when GFJ was combined with metformin (8.31 ± 3.48 mmol/L), suggesting that dietary modulation may still exacerbate adverse effects by altering drug distribution and clearance in the liver [193].

Beyond GFJ, other citrus bioactives may also impact CYP3A4 activity. Narirutin, a flavanone found abundantly in oranges and related citrus species, has been evaluated through in silico and in vitro models for its capacity to interact with multiple diabetes-related targets. While its main actions are directed toward inhibiting α-glucosidase and α-amylase, molecular docking studies suggest that narirutin may also bind to CYP3A4, raising the possibility of a competitive inhibitory effect similar to that of other citrus flavonoids such as naringenin [194].

Furthermore, Eriomin^®^, a citrus-derived supplement composed of a mixture of bioactive flavonoids, has demonstrated clinically significant improvements in glycemic control and systemic inflammation. In a double-blind, crossover trial in subjects with hyperglycemia, Eriomin supplementation (200 mg/day for 12 weeks) led to reductions in fasting glucose (−5%), HOMA-IR (−11%), and proinflammatory cytokines such as IL-6 (−14%) and TNF-α (−20%), alongside a 17% increase in GLP-1 concentrations. Although the primary mechanism of action appears to be anti-inflammatory and incretin-mediated, it is plausible that CYP3A4 inhibition contributes to enhanced incretin hormone activity or slowed metabolism of other co-administered agents, a hypothesis that warrants further study [195].

A broader pharmacokinetic study including glibenclamide under microdosing conditions also demonstrated that GFJ significantly reduced systemic exposure to several orally administered substrates, particularly those dependent on intestinal absorption and CYP3A4 metabolism. While atorvastatin (a known OATP2B1 inhibitor) had no such effect, GFJ decreased glibenclamide exposure more markedly, suggesting that citrus compounds may influence multiple absorption pathways, with CYP3A4 inhibition playing a central role [187].

Taken together, the current evidence strongly supports that dietary modulation of CYP3A4 by citrus constituents can significantly alter the pharmacokinetics of certain antidiabetic drugs, especially those with high intestinal metabolism such as repaglinide. While other drugs like glibenclamide and metformin are less directly affected, their pharmacological profiles may still be modulated indirectly by citrus-induced changes in hepatic or transporter-mediated disposition. These findings underscore the importance of evaluating dietary habits, particularly citrus product consumption, when prescribing CYP3A4-metabolized antidiabetic agents, to avoid undesired elevations in drug exposure or compromised glycemic control.

### 6.2. Polyphenols and Flavonoids

As previously described in Section 4, polyphenols and flavonoids modulate CYP3A4 activity through both inhibitory and inductive mechanisms [196]. The following section summarizes preclinical and clinical evidence on their impact on statin and antidiabetic drug pharmacokinetics.

In vitro studies have demonstrated that specific flavonoids, such as chrysin, quercetin, kaempferol, and leachianone A, can act as CYP3A4 inhibitors, reducing enzyme activity in a concentration-dependent manner [197]. These inhibitory effects are often linked to the structural arrangement of hydroxyl groups on the flavonoid backbone, which influences binding affinity to the enzyme’s active site. For instance, chrysin has shown potent inhibition with low IC50 values, indicating strong interaction potential [198].

In addition to structural considerations, flavonoid-mediated inhibition may differ depending on the experimental model. While previous sections have highlighted the inhibitory role of grapefruit flavonoids such as bergamottin and naringenin in human and rat microsomes (see Section 6.1), flavonoids also show significant inhibition in more physiologically relevant systems. For example, in primary rat hepatocytes, flavonoids reduced the intrinsic clearance of simvastatin more profoundly than in microsomal preparations, supporting the relevance of cellular models in predicting in vivo interactions [181].

Beyond inhibition, polyphenols may also influence CYP3A4 gene expression. Preclinical animal models have demonstrated that polyphenol-rich diets can alter CYP3A4 activity in hepatic and intestinal tissues, leading to changes in drug metabolism [196,199]. These interactions may lead to altered pharmacokinetics of co-administered drugs, including increased systemic exposure or reduced clearance, depending on the nature of the flavonoid and the drug involved. Additionally, the role of gut microbiota in metabolizing polyphenols adds another layer of complexity, as microbial biotransformation can influence both the bioavailability and the inhibitory potential of these compounds [200].

Clinical studies, although more limited, have begun to explore the relevance of these findings in humans. The most notable example is the GFJ effect, where flavonoids such as naringenin and bergamottin inhibit intestinal CYP3A4, leading to increased plasma concentrations of drugs like simvastatin and felodipine [201]. Similar interactions have been observed with flavonoid-rich supplements, raising concerns about food-drug interactions in patients undergoing pharmacotherapy [202].

Overall, both preclinical and clinical evidence support the notion that polyphenolic flavonoids can modulate CYP3A4 activity, with implications for drug metabolism, efficacy, and safety. These findings underscore the importance of considering dietary habits and supplement use in pharmacokinetic assessments and personalized medicine strategies.

### 6.3. Herbal Supplements

#### 6.3.1. St. John’s Wort

Among the most well-characterized herbal modulators of CYP450 enzymes, *Hypericum perforatum* (St. John’s Wort, SJW) stands out as a potent and clinically significant inducer of CYP3A4 via activation of the PXR [73]. Its primary active constituent, hyperforin, binds to PXR and upregulates the expression of several drug-metabolizing enzymes and transporters, including CYP3A4, CYP2C9, CYP2C19, and ABCB1 (P-glycoprotein) [73]. This mechanism, comparable in magnitude to rifampicin [72,203], has been confirmed in both in vitro models and clinical trials.

Pharmacokinetic studies have demonstrated that administration of standardized SJW extracts for 10–14 days significantly reduces the systemic exposure of several CYP3A4 substrates, including midazolam, simvastatin, and cyclosporine, with AUC decreases often exceeding 50% [204,205,206]. These reductions are attributed to combined hepatic and intestinal CYP3A4 induction and enhanced drug efflux via P-glycoprotein upregulation. Clinically, these interactions can be severe. In transplant patients, SJW has been associated with subtherapeutic cyclosporine levels and episodes of acute graft rejection [207].

The induction of CYP3A4 and other drug-metabolizing enzymes or transporters by SJW is both dose- and time-dependent. In vitro studies show that higher concentrations and longer exposure to *H. perforatum* or its active component hyperforin lead to greater induction of CYP3A4 mRNA expression, with effects varying by cell type and duration of treatment [208]. Clinical and preclinical evidence also indicates that continuous and high-dose use of H. perforatum results in stronger enzyme induction, which can significantly impact the metabolism of co-administered drugs [208,209]. Induction of other enzymes such as CYP2C9, CYP2C19, and transporters like P-glycoprotein is also observed, though typically to a lesser extent than CYP3A4, and these effects are similarly dose- and time-dependent [208,210]. Importantly, the enzyme-inducing effects of H. perforatum do not stop immediately after discontinuation; the increased activity of CYP3A4 and P-glycoprotein can persist for several days to weeks, depending on the duration and dose of prior use, as the body gradually returns to baseline enzyme levels [209]. This persistence means that drug interactions may continue even after stopping St. John’s Wort, and careful monitoring is advised when switching or discontinuing therapy.

The clinical implications are particularly relevant for commonly prescribed drugs such as statins and pioglitazone. A 14-day regimen of SJW reduced simvastatin AUC by over 50%, potentially compromising its lipid-lowering effect [10,205]. Although pioglitazone is primarily metabolized by CYP2C8, CYP3A4 contributes to secondary metabolism [10]; thus, induction of this pathway may be especially problematic in individuals with pharmacogenetic variability [211,212]. Unlike CYP inhibition, enzyme induction is often clinically silent, which increases the risk of unnoticed therapeutic failure, unnecessary dose escalation, or disease progression [72,205].

The extent of CYP3A4 induction by *H. perforatum* is largely determined by the hyperforin content of the preparation, with higher concentrations resulting in stronger activation of the PXR and greater transcriptional upregulation of CYP3A4 and P-glycoprotein [213,214,215]. Both in vitro and clinical studies support a dose-dependent relationship between hyperforin levels and the reduction in systemic exposure to CYP3A4 substrates, with decreases in AUC reaching up to 79% in some high-hyperforin products [72,214,215]. The variability in hyperforin content across commercial supplements—exceeding a 60-fold range—poses a significant challenge to predicting interaction risk. Consequently, patients consuming SJW products with elevated hyperforin levels face a heightened risk of therapeutic failure when taking critical medications, including immunosuppressants, statins, or other agents commonly prescribed in midlife and older women [216,217]. Due to the variability in hyperforin content, several authors recommend clearer labeling and the preferential use of low- or zero-hyperforin *H. perforatum* formulations to reduce interaction risks. Regulatory inconsistencies further hinder safety monitoring, emphasizing the need for harmonized guidelines and greater clinical awareness [218].

In summary, there is robust evidence that SJW, through hyperforin-mediated PXR activation, is a strong and sustained inducer of CYP3A4 and P-glycoprotein. Its widespread use, frequent non-disclosure by patients, and capacity to lower systemic drug concentrations necessitate vigilance by healthcare professionals. Standardized preparations at therapeutic doses are sufficient to trigger relevant interactions, especially with narrow-therapeutic-window medications. For these reasons, SJW is contraindicated in numerous clinical guidelines for patients receiving essential pharmacotherapies [206,219,220]

#### 6.3.2. *Schisandra chinensis*

*Schisandra chinensis* is a traditional medicinal plant whose lignan constituents display complex modulatory effects on cytochrome P450 enzymes, particularly CYP3A4. Its main bioactive compounds—such as schisandrin A, B, C, gomisin A, B, C, G, and schisandrol A—belong to the dibenzocyclooctadiene class of lignans, known to influence both the expression and activity of drug-metabolizing enzymes through diverse mechanisms [221].

Some lignans in *S. chinensis*, including schisandrin A and B and gomisin B, have shown the ability to activate the pregnane X receptor (PXR), a nuclear receptor that regulates the transcription of genes encoding CYP3A4. In animal models, this activation has been associated with increased hepatic expression of Cyp3a, suggesting a potential for transcriptional upregulation under specific conditions [221]. However, such findings are primarily derived from rodent studies, and their extrapolation to humans remains uncertain.

In contrast, several lignans demonstrate potent direct inhibition of CYP3A4 catalytic activity. For instance, gomisin C and gomisin G significantly inhibited midazolam 1′-hydroxylation, nifedipine oxidation, and testosterone 6β-hydroxylation in recombinant human enzyme systems, with estimated increases in drug exposure (AUC) ranging from 8% to over 3000% in predictive models [222]. These inhibitory effects were substrate-specific and structurally dependent, influenced by the position of methylenedioxy groups on the lignan backbone.

Further supporting this inhibitory profile, gomisin A was shown to cause time-dependent inactivation of CYP3A4, attributed to the formation of reactive carbene metabolites that covalently bind to the enzyme’s active site. This mechanism-based inhibition was demonstrated in microsomal systems using glutathione as a trapping agent, confirming irreversible enzyme modification [223]. Additionally, gomisin A and schisandrol A inhibited CYP3A4 in a cell-free assay with IC_50_ values of 1.39 µM and 32.0 µM, respectively [224].

In vivo studies in rats have further illustrated this duality. Both unprocessed and vinegar-processed *S. chinensis* extracts altered CYP activity depending on dose and duration. Multiple-dose administration led to increased activity of some CYP isoforms, including CYP3A, suggesting the possibility of induction through PXR activation. Yet, inhibition of other isoforms was also observed, underscoring the complexity of whole-extract effects and the influence of preparation method [225].

Altogether, *Schisandra chinensis* exhibits a multifaceted pharmacological interaction with CYP3A4 that cannot be reduced to a single direction of effect. While certain lignans may activate gene expression via nuclear receptor pathways, others act as potent inhibitors of CYP3A4 activity, often in a time- and concentration-dependent manner. The net effect is likely context-dependent, influenced by the lignan profile, extract standardization, dosage, and duration of intake.

#### 6.3.3. *Ginkgo biloba*

*Ginkgo biloba* is one of the most widely consumed herbal supplements worldwide, especially among older adults and individuals seeking to improve cognitive function, memory, or mood. However, despite its natural origin, Ginkgo biloba extract (GBE) may influence the metabolism of co-administered drugs, primarily through the modulation of cytochrome P450 enzymes, particularly CYP3A4 [226]. The question of whether chronic use of GBE is capable of inducing CYP3A4 to a clinically relevant extent remains a subject of ongoing investigation. Current evidence highlights a complex and sometimes contradictory profile of interaction, shaped by factors such as dose, extract composition, treatment duration, and the pharmacokinetic properties of the concomitant medication.

Some clinical studies have demonstrated that GBE can exert inductive effects on CYP3A4. In a randomized open-label trial, Robertson et al. [227] administered standardized GBE (120 mg twice daily) to healthy volunteers for 14 days. The authors observed a significant reduction in the AUC and (Cmax of midazolam—a well-established CYP3A4 probe—by approximately 34% and 31%, respectively. These findings suggest that GBE may induce CYP3A metabolism, potentially increasing the clearance of drugs metabolized by this isoenzyme. However, it is noteworthy that no significant changes were found in the exposure to lopinavir or ritonavir, possibly due to the potent inhibitory effect of ritonavir on CYP3A4, which could have masked the inductive action of GBE. This highlights the importance of considering drug–drug interactions in polypharmacy settings and the variability of enzyme modulation depending on the pharmacological context.

In contrast, other studies have reported opposing effects, raising doubts about the consistency of CYP3A4 induction by GBE. In a 28-day study using a higher daily dose of GBE (360 mg/day), Uchida et al. [228] found that GBE increased the AUC of midazolam by 25% and reduced its oral clearance by 26%. These results are more consistent with CYP3A4 inhibition rather than induction, suggesting a net inhibitory effect under these experimental conditions. Such discrepancies between studies may be explained by differences in extract composition, duration of administration, or even ethnic variability in metabolism, as this latter study was conducted in a homogeneous Japanese population.

These conflicting results underscore the complex pharmacokinetic behavior of GBE, which appears to exhibit bidirectional effects on CYP3A4, possibly through the simultaneous presence of flavonoids and terpene lactones that have opposite activities in vitro. This mechanistic explanation is supported by Wang et al. [136], who identified biflavones such as ginkgetin and isoginkgetin as potent CYP3A4 inhibitors in vitro, suggesting that specific polyphenolic constituents could exert significant inhibitory effects depending on concentration and context.

Supporting this view, Unger [226] conducted a comprehensive review of both preclinical and clinical data and concluded that standardized extracts such as EGb 761, when used at recommended doses up to 240 mg/day, do not result in clinically relevant alterations in the metabolism of CYP3A4 substrates. In vitro studies often report enzyme induction or inhibition using non-physiological concentrations or unstandardized extracts, limiting their applicability to real-world use. However, at doses exceeding the standard therapeutic range—or with poorly standardized commercial preparations—there may still be a risk of interaction.

More recently, Zadoyan et al. [229] conducted a randomized crossover trial specifically designed to evaluate the interaction between EGb 761 and CYP3A4 using midazolam as a pharmacokinetic probe. After 14 days of EGb 761 intake at 240 mg/day, the authors reported no significant changes in the pharmacokinetic profile of midazolam, further confirming that standardized GBE is unlikely to induce CYP3A4 to a clinically relevant extent. This finding is particularly relevant for practitioners, as it suggests that GBE does not compromise the efficacy or safety of medications metabolized by this pathway under standard dosing conditions.

In conclusion, while Ginkgo biloba extract has the potential to modulate CYP3A4 activity, the clinical significance of such interactions appears limited when using standardized preparations at recommended doses. However, caution may still be warranted in certain scenarios: for instance, in patients receiving drugs with a narrow therapeutic index, or when GBE is used at high doses or in non-standardized formulations. Moreover, given the inconsistent results across studies, further research is needed to clarify the molecular mechanisms underlying GBE’s dual action on CYP3A4 and to determine whether genetic polymorphisms or specific patient populations may be more susceptible to its effects.

#### 6.3.4. *Ginseng*

Among the wide array of herbal products commonly used in traditional medicine and dietary supplements, *Panax ginseng* has received notable attention for its capacity to modulate cytochrome P450 enzymes, particularly CYP3A4. This enzyme, predominantly expressed in the liver and small intestine, is responsible for the metabolism of approximately 50–60% of all clinically used drugs. Understanding how herbal constituents influence CYP3A4 activity is crucial for anticipating herb–drug interactions, which can result in altered drug exposure, therapeutic failure, or toxicity.

In the case of *Panax ginseng*, the current body of evidence highlights a dual and somewhat contradictory profile regarding its regulatory impact on CYP3A4. On one hand, several in vitro studies have shown that specific ginsenosides, such as ginsenoside Rf and panaxytriol, can activate the PXR. For instance, panaxytriol, a polyacetylene alcohol and one of the major active components in red ginseng and Shenmai injection, has been found to significantly increase both mRNA and protein expression levels of PXR and CYP3A4 in HepG2 cells in a time- and concentration-dependent manner. This effect was confirmed in human PXR-overexpressing HepG2 cells and involved enhanced recruitment of coactivators such as steroid receptor coactivator-1 (SRC-1) and the acetyltransferase P300, leading to increased binding of the PXR-RXR complex to DNA response elements ER-6 and DR-3 within the CYP3A4 promoter region [230]. These findings were supported by dual-luciferase reporter gene assays demonstrating a robust transcriptional activation of CYP3A4 following panaxytriol exposure [231].

However, this apparent inductive potential does not consistently translate to in vivo or clinical settings. Contradictory evidence from animal studies suggests that *Panax ginseng* may instead downregulate CYP3A4 expression under certain conditions. In a well-designed experiment in rats, oral administration of *P. ginseng* extract for 7 consecutive days led to a significant reduction in the clearance of midazolam—a well-established CYP3A4 substrate—and was associated with decreased gene and protein expression of both CYP3A4 and PXR in liver tissues [232]. This suggests that ginseng may suppress hepatic CYP3A activity in vivo, potentially through feedback regulation or complex interactions between ginsenosides and nuclear receptors.

Clinical studies further complicate the picture. Anderson et al. [233] investigated whether *Panax ginseng* supplementation at a typical dose (100 mg twice daily for 14 days) could induce CYP3A activity in healthy volunteers. Using the urinary 6-β-hydroxycortisol/cortisol ratio—a validated non-invasive biomarker for CYP3A4 induction—they found no significant change after supplementation, indicating a lack of clinically meaningful effect on enzyme activity at standard doses. These human data contrast with the in vitro findings and suggest that despite the molecular capacity of certain ginseng constituents to engage CYP3A4 regulatory pathways, the net effect in vivo is minimal or potentially negligible at conventional dosing.

Moreover, the direction and magnitude of CYP3A4 modulation by ginseng may be influenced by multiple variables, including the specific type and concentration of extract, duration of administration, interindividual genetic differences in PXR or CAR expression, and the presence of co-administered substances. For instance, some studies suggest that the CAR, which shares overlapping regulatory functions with PXR, can antagonize PXR-mediated CYP3A4 induction in the presence of ginsenosides, further modulating the final enzymatic response [234].

Taken together, while mechanistic and preclinical evidence indicates that *Panax ginseng*—particularly through panaxytriol—can modulate CYP3A4 expression via PXR activation, this effect appears to be highly context-dependent and not consistently reproduced in clinical studies. The overall body of evidence suggests that *Panax ginseng* should not be classified as a reliable CYP3A4 inducer or inhibitor in clinical practice. Its use at standard doses in herbal formulations or dietary supplements is unlikely to cause significant alterations in CYP3A4-mediated drug metabolism, although caution may still be warranted in scenarios involving high-dose or long-term consumption, or when used alongside medications with a narrow therapeutic index.

For clarity and ease of comparison, Table 2 provides a concise summary of the pharmacokinetic characteristics and CYP3A4-related interactions of the agents discussed, including key preclinical and clinical findings and corresponding references.

## 7. Clinical Implications and Practical Recommendations Derived from CYP3A4 Genetic Variability

Genetic polymorphisms in the CYP3A4 enzyme substantially contribute to interindividual variability in the metabolism of numerous drugs, particularly statins and oral antidiabetic agents. Given its role as one of the most active isoforms within the cytochrome P450 family, CYP3A4 is central to the biotransformation of a wide range of xenobiotics and endogenous compounds. Its activity is modulated not only by environmental factors, such as dietary inhibitors (e.g., GFJ, myricetin) or inducers (e.g., rifampicin), but also by specific genetic variants that alter expression levels or enzymatic function [235,236].

Moreover, from a clinical perspective, identifying and understanding these polymorphisms is critical. Among the most clinically relevant alleles, *CYP3A4*22, *1B*, *1G*, and *4* have been consistently associated with altered pharmacokinetics and risk of adverse drug reactions. For instance, *CYP3A4*22 significantly reduces hepatic expression and enzymatic activity. In a pivotal multi-ethnic study, Wang et al. [237] demonstrated that carriers of *CYP3A4*22 required lower doses of atorvastatin, simvastatin, and lovastatin due to reduced metabolic clearance. In line with these results, similar observations were reported by Elalem et al. [238] in a Saudi cohort, where individuals carrying both *CYP3A4*22 and *CYP3A5*3 exhibited enhanced response to simvastatin, likely due to increased systemic exposure.

These findings support the recommendation that clinicians consider pharmacogenetic testing, particularly of the *CYP3A4*22 allele, in patients with statin intolerance or high sensitivity to standard dosing, as such individuals may benefit from reduced dosages to prevent toxicity [238,239].

In East Asian populations, the *CYP3A4*1G allele has received special attention. Gao [240] found that this variant was associated with improved lipid-lowering efficacy of atorvastatin but had no significant impact on simvastatin response. These data suggest that the impact of genetic variability is not uniform across substrates. Therefore, there is a need for drug-specific genotype-guided dosing recommendations [241,242]. In practical terms, this implies that genetic testing results should be interpreted in the context of the specific drug prescribed, avoiding generalizations across all statins. As a practical alternative, where possible, alternative statins less dependent on CYP3A4 metabolism (e.g., pravastatin, rosuvastatin) may be considered in patients with uncertain or complex genotypes.

Dietary habits may further modulate these genetic effects. In individuals with reduced-function CYP3A4 alleles, ingestion of inhibitors such as GFJ can amplify the risk of drug toxicity by further reducing metabolic capacity [243,244]. Therefore, nutritional counseling should accompany pharmacogenetic assessments, especially when prescribing CYP3A4-metabolized agents [93,245].

Beyond individual-level variability, population-level differences in allele frequency must also be considered in practice. Elalem et al. and Gao et al. [238,240] highlighted that the distribution of CYP3A4 variants varies markedly among ethnic groups, potentially explaining observed disparities in statin efficacy and toxicity. Consequently, regional or ethnicity-tailored pharmacogenomic approaches may improve therapeutic outcomes and equity in healthcare [246]. In ethnically diverse settings, incorporating ancestry-based pharmacogenomic algorithms into electronic health records may facilitate stratified prescribing practices and reduce the risk of under- or over-treatment [246].

In Latin American populations, Alvarado et al. [247] emphasized that individuals harboring both *CYP3A4* and *SLCO1B1* risk alleles have a markedly higher susceptibility to simvastatin- and atorvastatin-induced myopathy and intolerance. This synergy between transporters and metabolizing enzymes reinforces the need for multigene panels instead of single-gene testing when tailoring treatment regimens [248]. In clinical scenarios, for patients presenting with statin-related muscle symptoms, multigene testing allows identification of combined genetic risks and supports safer medication changes or dosing adjustments. Inclusion of both metabolizing enzymes and transporters is especially relevant in primary care and cardiology protocols [249].

In the context of type 2 diabetes, Sivadas et al. [250] analyzed over 1000 Indian genomes and described a distinctive distribution of *CYP3A4* and *CYP3A5* variants that may predispose to clinically significant drug–drug and gene–drug interactions involving non-insulin antidiabetic drugs (NIADs). Based on these findings, this evidence supports the implementation of local pharmacogenomic databases and integration of genetic profiles into clinical decision-making tools, particularly in populations with high prevalence of diabetes and polypharmacy [251].

While these findings are promising, however, it is important to note that not all studies report strong associations between CYP3A4 genotypes and clinical outcomes. Some investigations have shown minimal or inconsistent effects, likely due to the influence of other genes (e.g., *SLCO1B1*, *CYP3A5*), dietary exposures, or concomitant medications [252,253]. These findings underscore the need for integrative approaches that combine genetic, nutritional, and clinical data to achieve true precision medicine [252,253]. Hence, routine clinical implementation should be guided by comprehensive pharmacogenomic panels, ideally integrated with clinical decision support tools that consider environmental and lifestyle factors. Therefore, isolated genotyping, in the absence of contextual interpretation, may yield misleading or incomplete results.

In line with the genetic variability discussed in Section 4.3.1 and the microbiota-mediated modulation described in Section 4.3.2, patient-specific differences in CYP3A4 activity carry direct clinical significance. Gut microbiota composition and metabolic activity can influence CYP3A4 expression via metabolites such as short-chain fatty acids (e.g., butyrate), indoles, phenolic acids, and secondary bile acids, which modulate nuclear receptors (e.g., PXR, AhR) and may alter epigenetic regulation within the gut–liver axis [254,255,256]. In vivo, gut microbiota has been shown to affect midazolam metabolism in mice, with germ-free animals displaying significantly higher plasma AUC and tissue accumulation [257]. Clinical conditions or interventions that disrupt the microbiota—such as broad-spectrum antibiotic therapy, gastrointestinal disease, or major dietary changes—have been associated with altered pharmacokinetics of CYP3A4 substrates [258,259]. From a practical perspective, these findings underscore the importance of incorporating recent antibiotic exposure, gastrointestinal disorders, usual dietary patterns (including high fiber intake), and probiotic or supplement use into medication reviews. For instance, antibiotic-induced microbiota changes have been linked to disrupted drug metabolism and may warrant temporary dose adjustment or closer therapeutic drug monitoring [260]. While routine microbiome profiling is not yet standard practice, integrating microbiota-related considerations with pharmacogenetic data may better predict variable drug exposure, prevent adverse effects, and optimize therapeutic outcomes.

In conclusion, there is substantial evidence supporting the clinical utility of CYP3A4 genotyping, particularly for alleles *22*, *1B*, *1G*, and *4*, in optimizing the use of statins, especially simvastatin and atorvastatin. Dietary components such as GFJ, myricetin, or curcumin should be carefully evaluated in genetically predisposed individuals to avoid pharmacokinetic interactions. Although evidence on oral antidiabetic drugs is more limited, special attention is warranted when agents such as saxagliptin or glyburide are co-administered with dietary or pharmacological modulators. Overall, these insights support the implementation of pharmacogenetic testing, dietary screening, and patient education as part of routine clinical care to reduce adverse effects and enhance therapeutic efficacy.

## 8. Conclusions

The evidence reviewed highlights that dietary modulation of CYP3A4 is a complex, multifactorial process with important clinical implications for the safety and efficacy of commonly prescribed statins and antidiabetic drugs. A wide range of dietary constituents, including citrus furanocoumarins, polyphenols, vitamins, and herbal extracts, can inhibit or induce CYP3A4 activity through mechanisms involving direct enzyme interaction, nuclear receptor activation, and epigenetic regulation. These effects are further modulated by genetic polymorphisms, interindividual microbiota profiles, and the nutritional context in which these compounds are consumed. Such variability contributes to unpredictable changes in drug bioavailability, especially for drugs with a narrow therapeutic window or high first-pass metabolism. Importantly, while some food–drug interactions are well recognized (e.g., GFJ), others remain underappreciated in clinical practice despite growing mechanistic and experimental evidence. Given the increasing use of polypharmacy and dietary supplements, a more proactive approach is warranted to identify patients at risk and to implement preventive strategies. This may include patient education, careful drug selection, and closer monitoring when initiating or discontinuing CYP3A4-modulating dietary components. Future studies should prioritize integrated models that account for genetics, diet, microbiome, and lifestyle factors to support personalized drug therapy and minimize adverse interactions. Ultimately, bridging pharmacokinetics with nutrition science may offer new opportunities for precision medicine in chronic disease management.

## Figures and Tables

**Table 1:** CYP3A4 involvement and drug–drug interactions of selected statins and antidiabetic agents.

Drug	Class	CYP3A4 Role	Main PK Characteristics	CYP3A4-Related Interactions	Clinical Notes
Simvastatin	Statin (lipophilic)	Major metabolic pathway (hepatic and intestinal)	Prodrug, converted to active acid; bioavailability < 5%; half-life ≈ 1.9 h; mainly biliary excretion	Strong CYP3A4 inhibitors (ketoconazole, erythromycin, ritonavir) increase exposure and myopathy risk	Modified-release formulations increase exposure due to reduced intestinal metabolism
Atorvastatin	Statin (lipophilic)	Major pathway (hepatic and intestinal); lactone form has higher affinity	Bioavailability ≈ 14%; half-life ≈ 7 h; mainly biliary excretion; high protein binding	Grapefruit juice, itraconazole, and ritonavir increase exposure and myopathy/rhabdomyolysis risk	Glucuronidation (UGT1A1, UGT1A3) also contributes significantly
Lovastatin	Statin (lipophilic)	Major pathway (intestinal > hepatic)	Prodrug, converted to active acid; bioavailability < 5%; half-life ≈ 3 h; biliary excretion	CYP3A4 inhibitors (erythromycin, ketoconazole, grapefruit juice) increase exposure	Novel delivery systems aim to reduce intestinal metabolism
Cerivastatin	Statin (synthetic)	Minor role (CYP3A4 + CYP2C8)	High potency; half-life 2–3 h; no unchanged drug excreted	CYP2C8 inhibitors (gemfibrozil) markedly increase rhabdomyolysis risk; CYP3A4 inhibition may add effect	Withdrawn in several countries due to safety concerns
Repaglinide	Meglitinide	Secondary to CYP2C8	Rapid onset; half-life ≈ 1–1.5 h; hepatic metabolism	CYP2C8 inhibitors (gemfibrozil, clopidogrel metabolite) increase exposure; CYP3A4 inhibitors (clarithromycin) moderate effect; rifampicin decreases exposure	Narrow therapeutic window with risk of hypoglycemia
Nateglinide	Meglitinide	Secondary to CYP2C9	Rapid onset; half-life ≈ 1.5 h; hepatic metabolism	Potent CYP2C9 inhibitors increase exposure; CYP3A4 inhibitors have minor effect; rifampicin decreases exposure	Lower risk of prolonged hypoglycemia compared with repaglinide
Saxagliptin	DPP-4 inhibitor	Major pathway (CYP3A4/5)	Time to peak ≈ 4 h; active metabolite (M2)	CYP3A4 inhibitors increase exposure; inducers decrease exposure; grapefruit juice may enhance effect	Limited dietary interaction studies available
Canagliflozin	SGLT2 inhibitor	Minor pathway	Mainly metabolized by glucuronidation (UGT1A9, UGT2B4); dual excretion routes	Strong CYP3A4 inducers (rifampicin) decrease exposure	Dose adjustment may be required with inducers
Pioglitazone	TZD	Secondary to CYP2C8	CYP3A4 contributes ≈ 37% of metabolism; long half-life	CYP3A4 inhibitors slightly increase exposure; inducers decrease exposure	Greater CYP3A4 involvement if CYP2C8 is inhibited

CYP2C8: Cytochrome P450 2C8, CYP2C9: Cytochrome P450 2C9, CYP3A4: Cytochrome P450 3A4, CYP3A5: Cytochrome P450 3A5, DPP-4: Dipeptidyl peptidase-4, M2: Active metabolite (of saxagliptin), SGLT2: Sodium-glucose cotransporter-2, TZD: Thiazolidinedione, UGT1A1/1A3/1A9/2B4: UDP-glucuronosyltransferase isoforms.

**Table 2:** Summary of CYP3A4-related interactions from preclinical and clinical studies.

Compound/Source	Model	CYP3A4/Transporter Effect	Main PK/PD Outcomes	References
Grapefruit juice (GFJ) and bergamottin	Preclinical and human	Strong intestinal CYP3A4 inhibition	Statins (atorvastatin, simvastatin): increased AUC/Cmax up to 15-fold, decreased metabolites, and enhanced lipid-lowering effect	[178,179,182]
Bergamottin and naringenin	In vitro and hepatocytes	Mixed-type inhibition; naringenin stronger than bergamottin in rats	Decreased simvastatin clearance; inhibition constant (Ki) 4–34 μM	[180,181]
GFJ—differential statin effect	Human	CYP3A4-mediated PK changes vary by statin	Atorvastatin AUC increased 83%, pitavastatin increased 13%, pravastatin showed minimal effect	[183,184]
GFJ chronic use	Human	Mild increase in atorvastatin exposure; no relevant lipid or toxicity changes	AUC increased by 19–26%	[185]
GFJ timing effect	Human	Reversible inhibition depending on timing of intake	Simvastatin AUC increased 13.5-fold when co-administered immediately, 2.1-fold after 24 h, and no change after 3–7 days	[36]
GFJ—transporter effects	Human and animals	Inhibits OATP2B1 and OATP1A2, alters P-glycoprotein (P-gp)	Decreased exposure of fexofenadine, sulfasalazine, and aliskiren; altered diltiazem permeability	[186,187,188]
GFJ—other drugs	Human and animals	Variable effects on non-CYP3A4 drugs	Repaglinide showed a slight increase in AUC, glibenclamide showed no change, and metformin showed increased hepatic levels and lactic acid	[191,192,193]
Other citrus flavonoids	In vitro and human	Possible CYP3A4 inhibition	Narirutin predicted to bind to CYP3A4 active site; Eriomin^®^ associated with decreased glucose and inflammation	[194,195]
St. John’s Wort (*Hypericum perforatum*)	In vitro, animal and human	Strong CYP3A4 and P-gp induction via pregnane X receptor (PXR) activation	Decreased AUC of midazolam, simvastatin, and cyclosporine (reductions > 50%); increased risk of graft rejection; effect dependent on hyperforin content	[10,72,73,203,207,208,209,210,211,212,213,214]
*Schisandra chinensis*	In vitro, animal	Dual effect: some lignans induce CYP3A4 via PXR, others act as inhibitors (substrate-specific)	Gomisin A and G cause time-dependent inhibition; multi-dose extracts may lead to induction	[222,223,224,225]
Ginkgo biloba extract	In vitro, animal and human	Bidirectional effects, depending on dose and extract quality	Standardized EGb 761 (≤240 mg/day) showed no significant CYP3A4 change; high-dose or poorly standardized preparations may interact	[136,227,228,229]
*Panax ginseng*	In vitro, animal and human	Context-dependent PXR activation or CYP3A4 downregulation	In vitro studies show increased CYP3A4 mRNA and protein; animal and human studies indicate minimal or no effect at standard doses	[230,231,232,233,234]

GFJ: Grapefruit juice, AUC: Area Under the Curve, Cmax: Maximum Concentration, Ki: Inhibition Constant, mRNA: messenger RNA, OATP2B1/1A2: Organic Anion Transporting Polypeptides, P-gp: P-glycoprotein, PXR: Pregnane X Receptor, CYP3A4: Cytochrome P450 3A4, PK/PD: Pharmacokinetics/Pharmacodynamics, EGb 761: Standardized Ginkgo biloba extract.

## Data Availability

No new data were created or analyzed in this study. Data sharing is not applicable to this article.

## Abbreviations

The following abbreviations are used in this manuscript:

AhR	Aryl hydrocarbon Receptor
AUC	Area Under the Curve
CAR	Constitutive Androstane Receptor
Cmax	Maximum Plasma Concentration
CYP3A4	Cytochrome P450 Family 3 Subfamily A Member 4
CYP450	Cytochrome P450 Enzyme Family
DPP-4	Dipeptidyl Peptidase-4
EGCG	Epigallocatechin Gallate
EOs	Essential Oils
GFJ	Grapefruit Juice
GIP	Glucose-dependent Insulinotropic Polypeptide
GLP-1	Glucagon-like Peptide-1
HDAC	Histone Deacetylase
IPA	Indole-3-propionic Acid
LDL-C	Low-Density Lipoprotein Cholesterol
LPS	Lipopolysaccharides
miRNA	MicroRNA
NIADs	Non-Insulin Antidiabetic Drugs
PPARγ	Peroxisome Proliferator-Activated Receptor Gamma
PPREs	Peroxisome Proliferator Response Elements
PXR	Pregnane X Receptor
RXR	Retinoid X Receptor
SAM	S-adenosylmethionine
SGLT-2	Sodium–Glucose Cotransporter 2
SJW	St. John’s Wort
SRC-1	Steroid Receptor Coactivator-1
SUR1	Sulfonylurea Receptor 1
T2D	Type 2 Diabetes
VDR	Vitamin D Receptor

## References

[1] Guengerich F.P. (2008). Cytochrome P450 and Chemical Toxicology. Chem. Res. Toxicol..

[2] Wilkinson G.R. (2005). Drug Metabolism and Variability among Patients in Drug Response. N. Engl. J. Med..

[3] Daly A.K., Rettie A.E., Fowler D.M., Miners J.O. (2018). Pharmacogenomics of CYP2C9: Functional and Clinical Considerations. J. Pers. Med..

[4] Niemi M., Backman J.T., Neuvonen M., Neuvonen P.J. (2003). Effect of Rifampicin on the Pharmacokinetics and Pharmacodynamics of Nateglinide in Healthy Subjects. Br. J. Clin. Pharmacol..

[5] Prueksaritanont T., Vega J.M., Zhao J., Gagliano K., Kuznetsova O., Musser B., Amin R.D., Liu L., Roadcap B.A., Dilzer S. (2001). Interactions between Simvastatin and Troglitazone or Pioglitazone in Healthy Subjects. J. Clin. Pharmacol..

[6] Dayyih W.A., Ani I.A.-, Hailat M., Alarman S.M., Zakaraya Z., Assab M.A., Alkhader E. (2014). Review of Grapefruit Juice-Drugs Interactions Mediated by Intestinal CYP3A4 Inhibition. J. Appl. Pharm. Sci..

[7] Lown K.S., Bailey D.G., Fontana R.J., Janardan S.K., Adair C.H., Fortlage L.A., Brown M.B., Guo W., Watkins P.B. (1997). Grapefruit Juice Increases Felodipine Oral Availability in Humans by Decreasing Intestinal CYP3A Protein Expression. J. Clin. Investig..

[8] Paine M.F., Hart H.L., Ludington S.S., Haining R.L., Rettie A.E., Zeldin D.C. (2006). The human intestinal cytochrome P450 “pie”. Drug Metab. Dispos..

[9] Markowitz J.S., Donovan J.L., DeVane C.L., Taylor R.M., Ruan Y., Wang J.S., Chavin K.D. (2003). Effect of St John’s Wort on Drug Metabolism by Induction of Cytochrome P450 3A4 Enzyme. JAMA.

[10] Sugimoto K. (2001). Different Effects of St John’s Wort on the Pharmacokinetics of Simvastatin and Pravastatin. Clin. Pharmacol. Ther..

[11] Wang Z., Gorski J.C., Hamman M.A., Huang S.-M., Lesko L.J., Hall S.D. (2001). The Effects of St John’s Wort *(Hypericum perforatum*) on Human Cytochrome P450 Activity. Clin. Pharmacol. Ther..

[12] Lim S.H., Bae S., Lee H.S., Han H.-K., Choi C.-I. (2023). Effect of Betanin, the Major Pigment of Red Beetroot (*Beta vulgaris* L.), on the Activity of Recombinant Human Cytochrome P450 Enzymes. Pharmaceuticals.

[13] Zhang M., Qiu Z. (2024). The Impact of Freeze-Dried Baiyedancong-Oolong Tea Aqueous Extract Containing Bioactive Compounds on the Activities of CYP450 Enzymes, the Transport Capabilities of P-Gp and OATs, and Transcription Levels in Mice. Food Nutr. Res..

[14] Basheer L., Kerem Z. (2015). Interactions between CYP3A4 and Dietary Polyphenols. Oxid. Med. Cell. Longev..

[15] Sadeghi S.J., Nardo G.D., Gilardi G. (2020). Chimeric Cytochrome P450 3A4 Used for In Vitro Prediction of Food–Drug Interactions. Biotechnol. Appl. Biochem..

[16] Fujino C., Sanoh S., Katsura T. (2021). Variation in Expression of Cytochrome P450 3A Isoforms and Toxicological Effects: Endo- and Exogenous Substances as Regulatory Factors and Substrates. Biol. Pharm. Bull..

[17] Burk O., Wojnowski L. (2004). Cytochrome P450 3A and Their Regulation. Naunyn. Schmiedebergs Arch. Pharmacol..

[18] Thummel K.E. (2007). Gut Instincts: CYP3A4 and Intestinal Drug Metabolism. J. Clin. Investig..

[19] Guengerich F.P. (2019). Cytochrome P450 Research and The Journal of Biological Chemistry. J. Biol. Chem..

[20] Neuvonen P.J., Niemi M., Backman J.T. (2006). Drug Interactions with Lipid-Lowering Drugs: Mechanisms and Clinical Relevance. Clin. Pharmacol. Ther..

[21] Kuehl P., Zhang J., Lin Y., Lamba J., Assem M., Schuetz J., Watkins P.B., Daly A., Wrighton S.A., Hall S.D. (2001). Sequence Diversity in CYP3A Promoters and Characterization of the Genetic Basis of Polymorphic CYP3A5 Expression. Nat. Genet..

[22] Kantola T., Kivistö K.T., Neuvonen P.J. (1998). Grapefruit Juice Greatly Increases Serum Concentrations of Lovastatin and Lovastatin Acid. Clin. Pharmacol. Ther..

[23] Zhou S.-F. (2008). Drugs Behave as Substrates, Inhibitors and Inducers of Human Cytochrome P450 3A4. Curr. Drug Metab..

[24] Pesaresi A. (2023). Mixed and Non-Competitive Enzyme Inhibition: Underlying Mechanisms and Mechanistic Irrelevance of the Formal Two-Site Model. J. Enzyme Inhib. Med. Chem..

[25] Guengerich F.P. (2019). Inhibition of Drug Metabolizing Enzymes. Handbook of Drug Metabolism.

[26] Kamel A., Harriman S. (2013). Inhibition of Cytochrome P450 Enzymes and Biochemical Aspects of Mechanism-Based Inactivation (MBI). Drug Discov. Today Technol..

[27] Zhou S., Yung Chan S., Cher Goh B., Chan E., Duan W., Huang M., McLeod H.L. (2005). Mechanism-Based Inhibition of Cytochrome P450 3A4 by Therapeutic Drugs. Clin. Pharmacokinet..

[28] Ogg M.S., Gray T.J., Gibson G.G. (1997). Development of an In Vitro Reporter Gene Assay to Assess Xenobiotic Induction of the Human CYP3A4 Gene. Eur. J. Drug Metab. Pharmacokinet..

[29] Seden K., Dickinson L., Khoo S., Back D. (2010). Grapefruit-Drug Interactions. Drugs.

[30] Yan J., Hirao H. (2025). QM/MM Study of the Metabolic Oxidation of 6′,7′-Dihydroxybergamottin Catalyzed by Human CYP3A4: Preferential Formation of the γ-Ketoenal Product in Mechanism-Based Inactivation. J. Chem. Inf. Model..

[31] Shalaby A.S., Eid H.H., El-Shiekh R.A., Mohamed O.G., Tripathi A., Al-Karmalawy A.A., Sleem A.A., Morsy F.A., Ibrahim K.M., Tadros S.H. (2023). Taming Food-Drug Interaction Risk: Potential Inhibitory Effects of Citrus Juices on Cytochrome Liver Enzymes Can Safeguard the Liver from Overdose Paracetamol-Induced Hepatotoxicity. ACS Omega.

[32] Fuhr L.M., Marok F.Z., Fuhr U., Selzer D., Lehr T. (2023). Physiologically Based Pharmacokinetic Modeling of Bergamottin and 6,7-Dihydroxybergamottin to Describe CYP3A4 Mediated Grapefruit-Drug Interactions. Clin. Pharmacol. Ther..

[33] Paine M.F., Criss A.B., Watkins P.B. (2004). Two Major Grapefruit Juice Components Differ in Intestinal CYP3A4 Inhibition Kinetic and Binding Properties. Drug Metab. Dispos..

[34] Paine M.F., Criss A.B., Watkins P.B. (2005). Two Major Grapefruit Juice Components Differ in Time to Onset of Intestinal CYP3A4 Inhibition. J. Pharmacol. Exp. Ther..

[35] Schmiedlin-Ren P., Edwards D.J., Fitzsimmons M.E., He K., Lown K.S., Woster P.M., Rahman A., Thummel K.E., Fisher J.M., Hollenberg P.F. (1997). Mechanisms of Enhanced Oral Availability of CYP3A4 Substrates by Grapefruit Constituents: Decreased Enterocyte CYP3A4 Concentration and Mechanism-Based Inactivation by Furanocoumarins. Drug Metab. Dispos..

[36] Lilja J.J., Kivistö K.T., Neuvonen P.J. (2000). Duration of Effect of Grapefruit Juice on the Pharmacokinetics of the CYP3A4 Substrate Simvastatin. Clin. Pharmacol. Ther..

[37] Takanaga H., Ohnishi A., Matsuo H., Murakami H., Sata H., Kuroda K., Urae A., Higuchi S., Sawada Y. (2000). Pharmacokinetic Analysis of Felodipine–Grapefruit Juice Interaction Based on an Irreversible Enzyme Inhibition Model. Br. J. Clin. Pharmacol..

[38] Greenblatt D.J., von Moltke L.L., Harmatz J.S., Chen G., Weemhoff J.L., Jen C., Kelley C.J., LeDuc B.W., Zinny M.A. (2003). Time Course of Recovery of Cytochrome P450 3A Function after Single Doses of Grapefruit Juice. Clin. Pharmacol. Ther..

[39] Melough M.M., Vance T.M., Lee S.G., Provatas A.A., Perkins C., Qureshi A., Cho E., Chun O.K. (2017). Furocoumarin Kinetics in Plasma and Urine of Healthy Adults Following Consumption of Grapefruit (*Citrus paradisi* Macf.) and Grapefruit Juice. J. Agric. Food Chem..

[40] Lundahl J., Regårdh C.G., Edgar B., Johnsson G. (1997). Effects of Grapefruit Juice Ingestion—Pharmacokinetics and Haemodynamics of Intravenously and Orally Administered Felodipine in Healthy Men. Eur. J. Clin. Pharmacol..

[41] Veronese M.L., Gillen L.P., Burke J.P., Dorval E.P., Hauck W.W., Pequignot E., Waldman S.A., Greenberg H.E. (2003). Exposure-Dependent Inhibition of Intestinal and Hepatic CYP3A4 In Vivo by Grapefruit Juice. J. Clin. Pharmacol..

[42] Edgar B., Bailey D., Bergstrand R., Johnsson G., Regårdh C.G. (1992). Acute Effects of Drinking Grapefruit Juice on the Pharmacokinetics and Dynamics of Felodipine—And Its Potential Clinical Relevance. Eur. J. Clin. Pharmacol..

[43] Wojtyniak J.-G., Selzer D., Schwab M., Lehr T. (2021). Physiologically Based Precision Dosing Approach for Drug-Drug-Gene Interactions: A Simvastatin Network Analysis. Clin. Pharmacol. Ther..

[44] Bailey D.G., Dresser G.K., Bend J.R. (2003). Bergamottin, Lime Juice, and Red Wine as Inhibitors of Cytochrome P450 3A4 Activity: Comparison with Grapefruit Juice. Clin. Pharmacol. Ther..

[45] Maneesai P., Jan-O B., Poasakate A., Rattanakanokchai S., Tong-Un T., Phuthong S., Pakdeechote P. (2023). Limonin Mitigates Cardiometabolic Complications in Rats with Metabolic Syndrome through Regulation of the IRS-1/GLUT4 Signalling Pathway. Biomed. Pharmacother. Biomed. Pharmacother..

[46] Qiu Y., Yang J., Ma L., Song M., Liu G. (2022). Limonin Isolated From Pomelo Seed Antagonizes Aβ25-35-Mediated Neuron Injury via PI3K/AKT Signaling Pathway by Regulating Cell Apoptosis. Front. Nutr..

[47] Chidambara Murthy K.N., Jayaprakasha G.K., Kumar V., Rathore K.S., Patil B.S. (2011). Citrus Limonin and Its Glucoside Inhibit Colon Adenocarcinoma Cell Proliferation through Apoptosis. J. Agric. Food Chem..

[48] Han Y.-L., Yu H.-L., Li D., Meng X.-L., Zhou Z.-Y., Yu Q., Zhang X.-Y., Wang F.-J., Guo C. (2011). Inhibitory Effects of Limonin on Six Human Cytochrome P450 Enzymes and P-Glycoprotein In Vitro. Toxicol. Vitro Int. J. Publ. Assoc. BIBRA.

[49] Paine M.F., Widmer W.W., Hart H.L., Pusek S.N., Beavers K.L., Criss A.B., Brown S.S., Thomas B.F., Watkins P.B. (2006). A Furanocoumarin-Free Grapefruit Juice Establishes Furanocoumarins as the Mediators of the Grapefruit Juice–Felodipine Interaction. Am. J. Clin. Nutr..

[50] Tresserra-Rimbau A., Lamuela-Raventos R.M., Moreno J.J. (2018). Polyphenols, Food and Pharma. Current Knowledge and Directions for Future Research. Biochem. Pharmacol..

[51] Kimura Y., Ito H., Ohnishi R., Hatano T. (2010). Inhibitory Effects of Polyphenols on Human Cytochrome P450 3A4 and 2C9 Activity. Food Chem. Toxicol..

[52] Vieira C.S.P., Freitas M., Palmeira A., Fernandes E., Araújo A.N. (2025). How Plant Polyhydroxy Flavonoids Can Hinder the Metabolism of Cytochrome 3A4. Biomedicines.

[53] Basheer L., Schultz K., Kerem Z. (2016). Inhibition of Cytochrome P450 3A by Acetoxylated Analogues of Resveratrol in In Vitro and in Silico Models. Sci. Rep..

[54] Sergent T., Dupont I., Heiden E.V.D., Scippo M.L., Pussemier L., Larondelle Y., Schneider Y.J. (2009). CYP1A1 and CYP3A4 Modulation by Dietary Flavonoids in Human Intestinal Caco-2 Cells. Toxicol. Lett..

[55] Hidaka M., Okumura M., Fujita K.-I., Ogikubo T., Yamasaki K., Iwakiri T., Setoguchi N., Arimori K. (2005). Effects of Pomegranate Juice on Human Cytochrome P450 3A (CYP3A) and Carbamazepine Pharmacokinetics in Rats. Drug Metab. Dispos. Biol. Fate Chem..

[56] Patil P.H., Birangal S., Shenoy G.G., Rao M., Kadari S., Wankhede A., Rastogi H., Sharma T., Pinjari J., Channabasavaiah J.P. (2022). Molecular Dynamics Simulation and In Vitro Evaluation of Herb–Drug Interactions Involving Dietary Polyphenols and CDK Inhibitors in Breast Cancer Chemotherapy. Phytother. Res..

[57] Tang H., Kuang Y., Wu W., Peng B., Fu Q. (2023). Quercetin Inhibits the Metabolism of Arachidonic Acid by Inhibiting the Activity of CYP3A4, Thereby Inhibiting the Progression of Breast Cancer. Mol. Med. Camb. Mass.

[58] Jimenez-Lopez J.M., Cederbaum A.I. (2004). Green Tea Polyphenol Epigallocatechin-3-Gallate Protects HepG2 Cells against CYP2E1-Dependent Toxicity. Free Radic. Biol. Med..

[59] Ji S.-B., Park S.-Y., Bae S., Seo H.-J., Kim S.-E., Lee G.-M., Wu Z., Liu K.-H. (2021). Comprehensive Investigation of Stereoselective Food Drug Interaction Potential of Resveratrol on Nine P450 and Six UGT Isoforms in Human Liver Microsomes. Pharmaceutics.

[60] Tsujimoto M., Agawa C., Ueda S., Yamane T., Kitayama H., Terao A., Fukuda T., Minegaki T., Nishiguchi K. (2017). Inhibitory Effects of Juices Prepared from Individual Vegetables on CYP3A4 Activity in Recombinant CYP3A4 and LS180 Cells. Biol. Pharm. Bull..

[61] Guthrie A.R., Chow H.-H.S., Martinez J.A. (2017). Effects of Resveratrol on Drug- and Carcinogen-Metabolizing Enzymes, Implications for Cancer Prevention. Pharmacol. Res. Perspect..

[62] Dresser G.K., Spence J.D., Bailey D.G. (2000). Pharmacokinetic-Pharmacodynamic Consequences and Clinical Relevance of Cytochrome P450 3A4 Inhibition. Clin. Pharmacokinet..

[63] Sang R., Jiang W., Zhang C., Yin R., Ouyang Z., Wei Y. (2025). Effect of Food Components on Cytochrome P450 Expression and Activity. Hum. Nutr. Metab..

[64] Deng R., Xu C., Chen X., Chen P., Wang Y., Zhou X., Jin J., Niu L., Ying M., Huang M. (2014). Resveratrol Suppresses the Inducible Expression of CYP3A4 through the Pregnane X Receptor. J. Pharmacol. Sci..

[65] Pannu N., Bhatnagar A. (2019). Resveratrol: From Enhanced Biosynthesis and Bioavailability to Multitargeting Chronic Diseases. Biomed. Pharmacother. Biomed. Pharmacother..

[66] Duda-Chodak A., Tarko T. (2023). Possible Side Effects of Polyphenols and Their Interactions with Medicines. Molecules.

[67] Li Y., Ning J., Wang Y., Wang C., Sun C., Huo X., Yu Z., Feng L., Zhang B., Tian X. (2018). Drug Interaction Study of Flavonoids toward CYP3A4 and Their Quantitative Structure Activity Relationship (QSAR) Analysis for Predicting Potential Effects. Toxicol. Lett..

[68] Bhardwaj R.K., Glaeser H., Becquemont L., Klotz U., Gupta S.K., Fromm M.F. (2002). Piperine, a Major Constituent of Black Pepper, Inhibits Human P-Glycoprotein and CYP3A4. J. Pharmacol. Exp. Ther..

[69] Poudel S., Huber A.D., Chen T. (2023). Regulation of Nuclear Receptors PXR and CAR by Small Molecules and Signal Crosstalk: Roles in Drug Metabolism and Beyond. Drug Metab. Dispos..

[70] Buchman C.D., Chai S.C., Chen T. (2018). A Current Structural Perspective on PXR and CAR in Drug Metabolism. Expert Opin. Drug Metab. Toxicol..

[71] Bettonte S., Berton M., Stader F., Battegay M., Marzolini C. (2023). Management of Drug Interactions with Inducers: Onset and Disappearance of Induction on Cytochrome P450 3A4 and Uridine Diphosphate Glucuronosyltransferase 1A1 Substrates. Eur. J. Drug Metab. Pharmacokinet..

[72] Hohmann N., Friedrichs A.S., Burhenne J., Blank A., Mikus G., Haefeli W.E. (2024). Dose-Dependent Induction of CYP3A Activity by St. John’s Wort Alone and in Combination with Rifampin. Clin. Transl. Sci..

[73] Moore L.B., Goodwin B., Jones S.A., Wisely G.B., Serabjit-Singh C.J., Willson T.M., Collins J.L., Kliewer S.A. (2000). St. John’s Wort Induces Hepatic Drug Metabolism through Activation of the Pregnane X Receptor. Proc. Natl. Acad. Sci. USA.

[74] Wentworth J.M., Agostini M., Love J., Schwabe J.W., Chatterjee V.K. (2000). St John’s Wort, a Herbal Antidepressant, Activates the Steroid X Receptor. J. Endocrinol..

[75] Xie H.-G., Kim R.B. (2005). St John’s Wort-Associated Drug Interactions: Short-Term Inhibition and Long-Term Induction?. Clin. Pharmacol. Ther..

[76] Gurley B.J., Gardner S.F., Hubbard M.A., Williams D.K., Gentry W.B., Cui Y., Ang C.Y.W. (2002). Cytochrome P450 Phenotypic Ratios for Predicting Herb-Drug Interactions in Humans. Clin. Pharmacol. Ther..

[77] Satyanarayana N.H., Visalakshmi V., Mukherjee S., Sarkar K.K. (2017). Studies on Genetic Diversity in Roselle (*Hibiscus sabdariffa* L.) for Seed Yield and It’s Contributing Traits in India. Vegetos.

[78] Srikanth Y., Reddy D.H., Anusha V.L., Dumala N., Viswanadh M.K., Chakravarthi G., Nalluri B.N., Yadagiri G., Ramakrishna K. (2025). Unveiling the Multifaceted Pharmacological Actions of Indole-3-Carbinol and Diindolylmethane: A Comprehensive Review. Plants.

[79] Vo Q.V., Mechler A. (2020). In Silico Study of the Radical Scavenging Activities of Natural Indole-3-Carbinols. J. Chem. Inf. Model..

[80] Anderton M.J., Manson M.M., Verschoyle R.D., Gescher A., Lamb J.H., Farmer P.B., Steward W.P., Williams M.L. (2004). Pharmacokinetics and Tissue Disposition of Indole-3-Carbinol and Its Acid Condensation Products after Oral Administration to Mice. Clin. Cancer Res. Off. J. Am. Assoc. Cancer Res..

[81] Pavek P., Dusek J., Smutny T., Lochman L., Kucera R., Skoda J., Smutna L., Kamaraj R., Soucek P., Vrzal R. (2022). Gene Expression Profiling of 1α,25(OH)2D3 Treatment in 2D/3D Human Hepatocyte Models Reveals CYP3A4 Induction but Minor Changes in Other Xenobiotic-Metabolizing Genes. Mol. Nutr. Food Res..

[82] Feierman D.E., Melinkov Z., Nanji A.A. (2003). Induction of CYP3A by Ethanol in Multiple In Vitro and In Vivo Models. Alcohol. Clin. Exp. Res..

[83] Raucy J.L. (2003). Regulation of CYP3A4 Expression in Human Hepatocytes by Pharmaceuticals and Natural Products. Drug Metab. Dispos..

[84] Qin X., Wang X. (2019). Role of Vitamin D Receptor in the Regulation of CYP3A Gene Expression. Acta Pharm. Sin. B.

[85] Bartonkova I., Dvorak Z. (2018). Essential Oils of Culinary Herbs and Spices Activate PXR and Induce CYP3A4 in Human Intestinal and Hepatic In Vitro Models. Toxicol. Lett..

[86] Minegishi G., Kobayashi Y., Fujikura M., Sano A., Kazuki Y., Kobayashi K. (2024). Induction of Hepatic CYP3A4 Expression by Cholesterol and Cholic Acid: Alterations of Gene Expression, Microsomal Activity, and Pharmacokinetics. Pharmacol. Res. Perspect..

[87] Klionsky D.J., Abdelmohsen K., Abe A., Abedin M.J., Abeliovich H., Acevedo Arozena A., Adachi H., Adams C.M., Adams P.D., Adeli K. (2016). Guidelines for the Use and Interpretation of Assays for Monitoring Autophagy (3rd Edition). Autophagy.

[88] Han C.J., Akowuah G.A., Shukkoor M.S.A., Biswas A. (2021). Modulation of Rat Hepatic Cyp3a4 Activity by Brassica Oleracea, Hibiscus Rosa Sinensis, and Tradescantia Zebrina. Biointerface Res. Appl. Chem..

[89] Murray M. (2005). Altered CYP Expression and Function in Response to Dietary Factors: Potential Roles in Disease Pathogenesis. Curr. Drug Metab..

[90] Drocourt L., Ourlin J.C., Pascussi J.M., Maurel P., Vilarem M.J. (2002). Expression of CYP3A4, CYP2B6, andCYP2C9 Is Regulated by the Vitamin D Receptor Pathway in Primary Human Hepatocytes. J. Biol. Chem..

[91] Montgomery M., Srinivasan A. (2019). Epigenetic Gene Regulation by Dietary Compounds in Cancer Prevention. Adv. Nutr..

[92] Prasad M., Rajagopal P., Devarajan N., Veeraraghavan V.P., Palanisamy C.P., Cui B., Patil S., Jayaraman S. (2022). A Comprehensive Review on High -Fat Diet-Induced Diabetes Mellitus: An Epigenetic View. J. Nutr. Biochem..

[93] Zarezadeh M., Saedisomeolia A., Shekarabi M., Khorshidi M., Emami M.R., Müller D.J. (2020). The Effect of Obesity, Macronutrients, Fasting and Nutritional Status on Drug-Metabolizing Cytochrome P450s: A Systematic Review of Current Evidence on Human Studies. Eur. J. Nutr..

[94] Liu R., Ding Y., Li W., Li S., Li X., Zhao D., Zhang Y., Wei G., Zhang X. (2023). Protective Role of Curcumin on Broiler Liver by Modulating Aflatoxin B1-Induced DNA Methylation and CYPs Expression. Ecotoxicol. Environ. Saf..

[95] Li D., Li Y., Yang S., Lu J., Jin X., Wu M. (2022). Diet-Gut Microbiota-Epigenetics in Metabolic Diseases: From Mechanisms to Therapeutics. Biomed. Pharmacother..

[96] Paul B., Barnes S., Demark-Wahnefried W., Morrow C., Salvador C., Skibola C., Tollefsbol T.O. (2015). Influences of Diet and the Gut Microbiome on Epigenetic Modulation in Cancer and Other Diseases. Clin. Epigenetics.

[97] Kumari A., Bhawal S., Kapila S., Yadav H., Kapila R. (2022). Health-Promoting Role of Dietary Bioactive Compounds through Epigenetic Modulations: A Novel Prophylactic and Therapeutic Approach. Crit. Rev. Food Sci. Nutr..

[98] Carlos-Reyes Á., López-González J.S., Meneses-Flores M., Gallardo-Rincón D., Ruíz-García E., Marchat L.A., Vega H.A.-D.L., Cruz O.N.H.D.L., López-Camarillo C. (2019). Dietary Compounds as Epigenetic Modulating Agents in Cancer. Front. Genet..

[99] Martino E., D’Onofrio N., Balestrieri A., Colloca A., Anastasio C., Sardu C., Marfella R., Campanile G., Balestrieri M.L. (2024). Dietary Epigenetic Modulators: Unravelling the Still-Controversial Benefits of miRNAs in Nutrition and Disease. Nutrients.

[100] Rubio K., Hernández-Cruz E.Y., Rogel-Ayala D.G., Sarvari P., Isidoro C., Barreto G., Pedraza-Chaverri J. (2023). Nutriepigenomics in Environmental-Associated Oxidative Stress. Antioxidants.

[101] Sundaram M.K., Preetha R., Haque S., Akhter N., Khan S., Ahmad S., Hussain A. (2022). Dietary Isothiocyanates Inhibit Cancer Progression by Modulation of Epigenome. Semin. Cancer Biol..

[102] Pavek P., Pospechova K., Svecova L., Syrova Z., Stejskalova L., Blazkova J., Dvorak Z., Blahos J. (2010). Intestinal Cell-Specific Vitamin D Receptor (VDR)-Mediated Transcriptional Regulation of CYP3A4 Gene. Biochem. Pharmacol..

[103] Zeng H., Lin Y., Gong J., Lin S., Gao J., Li C., Feng Z., Zhang H., Zhang J., Li Y. (2019). CYP3A Suppression during Diet-Induced Nonalcoholic Fatty Liver Disease Is Independent of PXR Regulation. Chem. Biol. Interact..

[104] Maggioni A.P., Calabria S., Rossi E., Martini N. (2017). Use of Lipid Lowering Drugs in Patients at Very High Risk of Cardiovascular Events: An Analysis on Nearly 3,000,000 Italian Subjects of the ARNO Observatory. Int. J. Cardiol..

[105] Chauvin B., Drouot S., Barrail-Tran A., Taburet A.M. (2013). Drug-Drug Interactions between HMG-CoA Reductase Inhibitors (Statins) and Antiviral Protease Inhibitors. Clin. Pharmacokinet..

[106] Mauro V.F. (1993). Clinical Pharmacokinetics and Practical Applications of Simvastatin. Clin. Pharmacokinet..

[107] Tubic-Grozdanis M., Hilfinger J.M., Amidon G.L., Kim J.S., Kijek P., Staubach P., Langguth P. (2008). Pharmacokinetics of the CYP 3A Substrate Simvastatin Following Administration of Delayed versus Immediate Release Oral Dosage Forms. Pharm. Res..

[108] Lennernäs H. (2003). Clinical Pharmacokinetics of Atorvastatin. Clin. Pharmacokinet..

[109] Hu N., Chen C., Wang J., Huang J., Yao D., Li C. (2021). Atorvastatin Ester Regulates Lipid Metabolism in Hyperlipidemia Rats via the PPAR-Signaling Pathway and HMGCR Expression in the Liver. Int. J. Mol. Sci..

[110] Stillemans G., Paquot A., Muccioli G.G., Hoste E., Panin N., Åsberg A., Balligand J., Haufroid V., Elens L. (2022). Atorvastatin Population Pharmacokinetics in a Real-life Setting: Influence of Genetic Polymorphisms and Association with Clinical Response. Clin. Transl. Sci..

[111] Cao M.-C., Huang X., Tang B.-H., Shi H.-Y., Zheng Y., Zhao W. (2024). Simultaneous Determination of the Combined and Free Concentrations of Atorvastatin and Its Major Metabolite In Vitro and In Vivo Based on Ultrafiltration Coupled with UPLC-MS/MS Method: An Application in a Protein Binding Rate and Metabolism Ability Study in Uremic Hemodialysis Patients. Front. Cardiovasc. Med..

[112] Hwang J.G., Yu K.-S., Lee S. (2020). Comparison of the Pharmacokinetics of Highly Variable Drugs in Healthy Subjects Using a Partial Replicated Crossover Study: A Fixed-Dose Combination of Fimasartan 120 Mg and Atorvastatin 40 Mg versus Separate Tablets. Drug Des. Devel. Ther..

[113] Reig-López J., García-Arieta A., Mangas-Sanjuán V., Merino-Sanjuán M. (2021). Current Evidence, Challenges, and Opportunities of Physiologically Based Pharmacokinetic Models of Atorvastatin for Decision Making. Pharmaceutics.

[114] Istvan E.S., Deisenhofer J. (2001). Structural Mechanism for Statin Inhibition of HMG-CoA Reductase. Science.

[115] Nf Z., Mmr M.M.A., Aba M. (2017). Lovastatin: History, Physicochemistry, Pharmacokinetics and Enhanced Solubility. Int. J. Res. Pharm. Sci..

[116] Singh S.K., Verma P.R.P., Razdan B. (2010). Development and Characterization of a Lovastatin-Loaded Self-Microemulsifying Drug Delivery System. Pharm. Dev. Technol..

[117] Bischoff H., Angerbauer R., Bender J., Bischoff E., Faggiotto A., Petzinna D., Pfitzner J., Porter M.C., Schmidt D., Thomas G. (1997). Cerivastatin: Pharmacology of a Novel Synthetic and Highly Active HMG-CoA Reductase Inhibitor. Atherosclerosis.

[118] Mück W. (2000). Clinical Pharmacokinetics of Cerivastatin. Clin. Pharmacokinet..

[119] Kaspera R., Naraharisetti S.B., Tamraz B., Sahele T., Cheesman M.J., Kwok P.-Y., Marciante K., Heckbert S.R., Psaty B.M., Totah R.A. (2010). Cerivastatin In Vitro Metabolism by CYP2C8 Variants Found in Patients Experiencing Rhabdomyolysis. Pharmacogenet. Genom..

[120] Marciante K.D., Durda J.P., Heckbert S.R., Lumley T., Rice K., McKnight B., Totah R.A., Tamraz B., Kroetz D.L., Fukushima H. (2011). Cerivastatin, Genetic Variants, and the Risk of Rhabdomyolysis. Pharmacogenet. Genom..

[121] Graham D.J., Staffa J.A., Shatin D., Andrade S.E., Schech S.D., La Grenade L., Gurwitz J.H., Chan K.A., Goodman M.J., Platt R. (2004). Incidence of Hospitalized Rhabdomyolysis in Patients Treated with Lipid-Lowering Drugs. JAMA.

[122] Ainurofiq A., Ismaya L. (2023). Pharmacokinetic Drug-Drug Interactions: A Systematic Review of the Cytochrome P450 (CYP) Isoenzyme 3A4. Res. J. Pharm. Technol..

[123] Tornio A., Niemi M., Neuvonen P.J., Backman J.T. (2012). Drug Interactions with Oral Antidiabetic Agents: Pharmacokinetic Mechanisms and Clinical Implications. Trends Pharmacol. Sci..

[124] Petrie J.R., Guzik T.J., Touyz R.M. (2018). Diabetes, Hypertension, and Cardiovascular Disease: Clinical Insights and Vascular Mechanisms. Can. J. Cardiol..

[125] Sheneni V.D., Shaibu I.E. (2023). Drug Interactions and Drug-Food Interactions in Patients Receiving Diabetes Mellitus Treatment. Endocrinol. Metab. Int. J..

[126] Jangra A., Babu B., Divakar S., Gowramma B., Rajan S., Jangra S., Malakar V. (2025). An In-Depth Review of PPARγ Modulators as Anti-Diabetes Therapeutics. Drug Metab. Rev..

[127] Graefe-Mody U., Retlich S., Friedrich C. (2012). Clinical Pharmacokinetics and Pharmacodynamics of Linagliptin. Clin. Pharmacokinet..

[128] Hwang I., Kim Y., Yoo H., Jang I.J., Yu K.S., Lee S. (2020). Pharmacokinetic/Pharmacodynamic Interaction Between Evogliptin and Pioglitazone in Healthy Male Subjects. Drug Des. Dev. Ther..

[129] Nowak S.N., Edwards D.J., Clarke A., Anderson G.D., Jaber L.A. (2002). Pioglitazone: Effect on CYP3A4 Activity. J. Clin. Pharmacol..

[130] Bidstrup T.B., Bjørnsdottir I., Sidelmann U.G., Thomsen M.S., Hansen K.T. (2003). CYP2C8 and CYP3A4 Are the Principal Enzymes Involved in the Human In Vitro Biotransformation of the Insulin Secretagogue Repaglinide. Br. J. Clin. Pharmacol..

[131] Jaakkola T., Laitila J., Neuvonen P.J., Backman J.T. (2006). Pioglitazone Is Metabolised by CYP2C8 and CYP3A4 In Vitro: Potential for Interactions with CYP2C8 Inhibitors. Basic Clin. Pharmacol. Toxicol..

[132] Mansour R.Y., ElBorolossy R., Shaheen S.M., Sabri N.A. (2022). Evaluation of Drug Interactions of Saxagliptin with Sildenafil in Healthy Volunteers. Eur. J. Clin. Pharmacol..

[133] Husain I., Manda V., Alhusban M., Dale O.R., Bae J.Y., Avula B., Gurley B.J., Chittiboyina A.G., Khan I.A., Khan S.I. (2021). Modulation of CYP3A4 and CYP2C9 Activity by Bulbine Natalensis and Its Constituents: An Assessment of HDI Risk of B. Natalensis Containing Supplements. Phytomedicine.

[134] Kennedy K.E., Teng C., Patek T.M., Frei C.R. (2020). Hypoglycemia Associated with Antibiotics Alone and in Combination with Sulfonylureas and Meglitinides: An Epidemiologic Surveillance Study of the FDA Adverse Event Reporting System (FAERS). Drug Saf..

[135] Parasrampuria D.A., Mendell J., Shi M., Matsushima N., Zahir H., Truitt K. (2016). Edoxaban Drug–Drug Interactions with Ketoconazole, Erythromycin, and Cyclosporine. Br. J. Clin. Pharmacol..

[136] Wang B., Shi C., Feng L., Pan W., Tian X.G., Sun C.P., Wang C., Ning J., Lv X., Wang Y. (2022). Potent Inhibition of Human Cytochrome P450 3A4 by Biflavone Components from Ginkgo Biloba and *Selaginella tamariscina*. Front. Pharmacol..

[137] Wanwimolruk S., Wong K., Wanwimolruk P. (2009). Variable Inhibitory Effect of Different Brands of Commercial Herbal Supplements on Human Cytochrome P-450 CYP3A4. Drug Metabol. Drug Interact..

[138] Gallo P., Vincentis A.D., Pedone C., Nobili A., Tettamanti M., Gentilucci U.V., Picardi A., Mannucci P.M., Incalzi R.A. (2019). Drug–Drug Interactions Involving CYP3A4 and p-Glycoprotein in Hospitalized Elderly Patients. Eur. J. Intern. Med..

[139] Klotz U. (2009). Pharmacokinetics and Drug Metabolism in the Elderly. Drug Metab. Rev..

[140] Tsujimoto M., Nagano Y., Hosoda S., Shiraishi A., Miyoshi A., Hiraoka S., Furukubo T., Izumi S., Yamakawa T., Minegaki T. (2013). Effects of Decreased Vitamin D and Accumulated Uremic Toxin on Human CYP3A4 Activity in Patients with End-Stage Renal Disease. Toxins.

[141] Scott L.J. (2012). Repaglinide: A Review of Its Use in Type 2 Diabetes Mellitus. Drugs.

[142] Ding D., Wang M., Wu J.X., Kang Y., Chen L. (2019). The Structural Basis for the Binding of Repaglinide to the Pancreatic KATP Channel. Cell Rep..

[143] Trexler A.J., Taraska J.W. (2017). Regulation of Insulin Exocytosis by Calcium-Dependent Protein Kinase C in Beta Cells. Cell Calcium.

[144] Guardado-Mendoza R., Prioletta A., Jiménez-Ceja L.M., Sosale A., Folli F. (2013). The Role of Nateglinide and Repaglinide, Derivatives of Meglitinide, in the Treatment of Type 2 Diabetes Mellitus. Arch. Med. Sci. AMS.

[145] Säll C.M.T. (2013). In Vitro-In Vivo Assessment of Repaglinide Metabolism and Drug-Drug Interactions:Towards a Physiologically-Based Pharmacokinetic Model.

[146] Säll C., Houston J.B., Galetin A. (2012). A Comprehensive Assessment of Repaglinide Metabolic Pathways: Impact of Choice of In Vitro System and Relative Enzyme Contribution to In Vitro Clearance. Drug Metab. Dispos..

[147] Akagi Y., Iketaki A., Kimura H., Matsudaira Y., Yoshida T., Nishimura T., Kawano Y., Mano Y., Shigematsu E., Ujihara M. (2020). Risk of Hypoglycemia Associated with Repaglinide Combined with Clopidogrel, a Retrospective Cohort Study. J. Pharm. Health Care Sci..

[148] Honkalammi J., Niemi M., Neuvonen P.J., Backman J.T. (2011). Dose-Dependent Interaction between Gemfibrozil and Repaglinide in Humans: Strong Inhibition of CYP2C8 with Subtherapeutic Gemfibrozil Doses. Drug Metab. Dispos..

[149] Bidstrup T.B., Stilling N., Damkier P., Scharling B., Thomsen M.S., Brøsen K. (2004). Rifampicin Seems to Act as Both an Inducer and an Inhibitor of the Metabolism of Repaglinide. Eur. J. Clin. Pharmacol..

[150] Lee S., Kim A.H.J., Yoon S., Lee J., Lee Y., Ji S.C., Yoon S.H., Lee S.H., Yu K.S., Jang I.J. (2021). The Utility of CYP3A Activity Endogenous Markers for Evaluating Drug-Drug Interaction between Sildenafil and CYP3A Inhibitors in Healthy Subjects. Drug Metab. Pharmacokinet..

[151] Kajosaari L.I., Laitila J., Neuvonen P.J., Backman J.T. (2005). Metabolism of Repaglinide by CYP2C8 and CYP3A4 In Vitro: Effect of Fibrates and Rifampicin. Basic Clin. Pharmacol. Toxicol..

[152] Hansen A.M.K., Christensen I.T., Hansen J.B., Carr R.D., Ashcroft F.M., Wahl P. (2002). Differential Interactions of Nateglinide and Repaglinide on the Human β-Cell Sulphonylurea Receptor 1. Diabetes.

[153] Krentz A. (2012). Established Classes of Glucose-Lowering Drugs. Drug Therapy for Type 2 Diabetes.

[154] Alanazi M.M. (2025). Nateglinide: A Comprehensive Profile. Profiles Drug Subst. Excip. Relat. Methodol..

[155] Weaver M.L., Orwig B., Rodriguez L., Graham E.D., Chin J., Shapiro M., McLeod J., Mangold J. (2001). Pharmacokinetics and Metabolism of Nateglinide in Humans. Drug Metab. Dispos..

[156] Maideen N.M.P., Manavalan G., Balasubramanian K. (2018). Drug Interactions of Meglitinide Antidiabetics Involving CYP Enzymes and OATP1B1 Transporter. Ther. Adv. Endocrinol. Metab..

[157] Asaumi R., Toshimoto K., Tobe Y., Hashizume K., Nunoya K.I., Imawaka H., Lee W., Sugiyama Y. (2018). Comprehensive PBPK Model of Rifampicin for Quantitative Prediction of Complex Drug-Drug Interactions: CYP3A/2C9 Induction and OATP Inhibition Effects. CPT Pharmacomet. Syst. Pharmacol..

[158] Takanohashi T., Koizumi T., Mihara R., Okudaira K. (2007). Prediction of the Metabolic Interaction of Nateglinide with Other Drugs Based on In Vitro Studies. Drug Metab. Pharmacokinet..

[159] Tentolouris N., Voulgari C., Katsilambros N. (2007). A Review of Nateglinide in the Management of Patients with Type 2 Diabetes. Vasc. Health Risk Manag..

[160] Dave D.J. (2011). Saxagliptin: A Dipeptidyl Peptidase-4 Inhibitor in the Treatment of Type 2 Diabetes Mellitus. J. Pharmacol. Pharmacother..

[161] Butrovich M.A., Tang W., Boulton D.W., Nolin T.D., Sharma P. (2022). Use of Physiologically Based Pharmacokinetic Modeling to Evaluate the Impact of Chronic Kidney Disease on CYP3A4-Mediated Metabolism of Saxagliptin. J. Clin. Pharmacol..

[162] Neumiller J.J. (2014). Efficacy and Safety of Saxagliptin as Add-On Therapy in Type 2 Diabetes. Clin. Diabetes.

[163] liu Q., Ou-Yang Q.G., Lin Q.M., Lu X.R., Ma Y.Q., Li Y.H., Xu R.A., Lin D., Hu G.X., Cai J.P. (2022). Effects of 27 CYP3A4 Protein Variants on Saxagliptin Metabolism In Vitro. Fundam. Clin. Pharmacol..

[164] Fura A., Khanna A., Vyas V., Koplowitz B., Chang S.Y., Caporuscio C., Boulton D.W., Christopher L.J., Chadwick K.D., Hamann L.G. (2009). Pharmacokinetics of the Dipeptidyl Peptidase 4 Inhibitor Saxagliptin in Rats, Dogs, and Monkeys and Clinical Projections. Drug Metab. Dispos..

[165] Patel C.G., Li L., Girgis S., Kornhauser D.M., Frevert E.U., Boulton D.W. (2011). Two-Way Pharmacokinetic Interaction Studies between Saxagliptin and Cytochrome P450 Substrates or Inhibitors: Simvastatin, Diltiazem Extended-Release, and Ketoconazole. Clin. Pharmacol. Adv. Appl..

[166] Chacra A.R. (2010). Saxagliptin for Type 2 Diabetes. Diabetes Metab. Syndr. Obes. Targets Ther..

[167] Government of Canada Summary Basis of Decision for Onglyza™. Drug and Health Product Portal. https://dhpp.hpfb-dgpsa.ca/review-documents/resource/SBD00107.

[168] McLean P., Bennett J., Chandrasekhar S., Newman N., Mohammad Y., Khawaja M., Rizwan A., Siddiqui R., Birnbaum Y., Edward “Trey” Woods (2025). SGLT2 Inhibitors across Various Patient Populations in the Era of Precision Medicine: The Multidisciplinary Team Approach. npj Metab. Health Dis..

[169] Deeks E.D., Scheen A.J. (2017). Canagliflozin: A Review in Type 2 Diabetes. Drugs.

[170] Neal B., Perkovic V., Mahaffey K.W., de Zeeuw D., Fulcher G., Erondu N., Shaw W., Law G., Desai M., Matthews D.R. (2017). Canagliflozin and Cardiovascular and Renal Events in Type 2 Diabetes. N. Engl. J. Med..

[171] Polidori D., Mari A., Ferrannini E. (2014). Canagliflozin, a Sodium Glucose Co-Transporter 2 Inhibitor, Improves Model-Based Indices of Beta Cell Function in Patients with Type 2 Diabetes. Diabetologia.

[172] Scheen A.J. (2015). Pharmacokinetics, Pharmacodynamics and Clinical Use of SGLT2 Inhibitors in Patients with Type 2 Diabetes Mellitus and Chronic Kidney Disease. Clin. Pharmacokinet..

[173] Mamidi R.N.V.S., Dallas S., Sensenhauser C., Lim H.K., Scheers E., Verboven P., Cuyckens F., Leclercq L., Evans D.C., Kelley M.F. (2016). In Vitro and Physiologically-based Pharmacokinetic Based Assessment of Drug–Drug Interaction Potential of Canagliflozin. Br. J. Clin. Pharmacol..

[174] Devineni D., Polidori D. (2015). Clinical Pharmacokinetic, Pharmacodynamic, and Drug–Drug Interaction Profile of Canagliflozin, a Sodium-Glucose Co-Transporter 2 Inhibitor. Clin. Pharmacokinet..

[175] Guengerich F.P. (2024). Roles of Individual Human Cytochrome P450 Enzymes in Drug Metabolism. Pharmacol. Rev..

[176] Shah M. (2025). Abstract P1039: Inhibition Of Drug Metabolizing Enzyme Cytochrome P450 2C8 By Acyl-Glucuronide Metabolites Of Gemfibrozil And Clopidogrel. Circulation.

[177] Forst T., Karagiannis E., Lübben G., Hohberg C., Schöndorf T., Dikta G., Drexler M., Morcos M., Dänschel W., Borchert M. (2008). Pleiotrophic and Anti-Inflammatory Effects of Pioglitazone Precede the Metabolic Activity in Type 2 Diabetic Patients with Coronary Artery Disease. Atherosclerosis.

[178] Zhang X., Guo J., Li J., Chen C., Su G. (2022). Pharmacokinetics and Pharmacodynamic Interaction of Bergamottin with Atorvastatin in Rats. Xenobiotica Fate Foreign Compd. Biol. Syst..

[179] Park S.-J., Yeo C.-W., Shim E.-J., Kim H., Liu K.-H., Shin J.-G., Shon J.-H. (2016). Pomegranate Juice Does Not Affect the Disposition of Simvastatin in Healthy Subjects. Eur. J. Drug Metab. Pharmacokinet..

[180] Le Goff-Klein N., Klein L., Hérin M., Koffel J.-C., Ubeaud G. (2004). Inhibition of In-Vitro Simvastatin Metabolism in Rat Liver Microsomes by Bergamottin, a Component of Grapefruit Juice. J. Pharm. Pharmacol..

[181] Ubeaud G., Hagenbach J., Vandenschrieck S., Jung L., Koffel J.C. (1999). In Vitro Inhibition of Simvastatin Metabolism in Rat and Human Liver by Naringenin. Life Sci..

[182] Lilja J.J., Kivistö K.T., Neuvonen P.J. (1999). Grapefruit Juice Increases Serum Concentrations of Atorvastatin and Has No Effect on Pravastatin. Clin. Pharmacol. Ther..

[183] Ando H., Tsuruoka S., Yanagihara H., Sugimoto K., Miyata M., Yamazoe Y., Takamura T., Kaneko S., Fujimura A. (2005). Effects of Grapefruit Juice on the Pharmacokinetics of Pitavastatin and Atorvastatin. Br. J. Clin. Pharmacol..

[184] Fukazawa I., Uchida N., Uchida E., Yasuhara H. (2004). Effects of Grapefruit Juice on Pharmacokinetics of Atorvastatin and Pravastatin in Japanese. Br. J. Clin. Pharmacol..

[185] Reddy P., Ellington D., Zhu Y., Zdrojewski I., Parent S.J., Harmatz J.S., Derendorf H., Greenblatt D.J., Browne K. (2011). Serum Concentrations and Clinical Effects of Atorvastatin in Patients Taking Grapefruit Juice Daily. Br. J. Clin. Pharmacol..

[186] Medwid S., Li M.M.J., Knauer M.J., Lin K., Mansell S.E., Schmerk C.L., Zhu C., Griffin K.E., Yousif M.D., Dresser G.K. (2019). Fexofenadine and Rosuvastatin Pharmacokinetics in Mice with Targeted Disruption of Organic Anion Transporting Polypeptide 2B1. Drug Metab. Dispos. Biol. Fate Chem..

[187] Kashihara Y., Ieiri I., Yoshikado T., Maeda K., Fukae M., Kimura M., Hirota T., Matsuki S., Irie S., Izumi N. (2017). Small-Dosing Clinical Study: Pharmacokinetic, Pharmacogenomic (SLCO2B1 and ABCG2), and Interaction (Atorvastatin and Grapefruit Juice) Profiles of 5 Probes for OATP2B1 and BCRP. J. Pharm. Sci..

[188] Rebello S., Zhao S., Hariry S., Dahlke M., Alexander N., Vapurcuyan A., Hanna I., Jarugula V. (2012). Intestinal OATP1A2 Inhibition as a Potential Mechanism for the Effect of Grapefruit Juice on Aliskiren Pharmacokinetics in Healthy Subjects. Eur. J. Clin. Pharmacol..

[189] Boddu S.P., Yamsani M.R., Potharaju S., Veeraraghavan S., Rajak S., Kuma S.V.V.S., Avery B.A., Repka M.A., Varanasi V.S.K.K. (2009). Influence of Grapefruit Juice on the Pharmacokinetics of Diltiazem in Wistar Rats upon Single and Multiple Dosage Regimens. Int. J. Pharm. Sci..

[190] Rogers J.D., Zhao J., Liu L., Amin R.D., Gagliano K.D., Porras A.G., Blum R.A., Wilson M.F., Stepanavage M., Vega J.M. (1999). Grapefruit Juice Has Minimal Effects on Plasma Concentrations of Lovastatin-Derived 3-Hydroxy-3-Methylglutaryl Coenzyme A Reductase Inhibitors. Clin. Pharmacol. Ther..

[191] Bidstrup T.B., Damkier P., Olsen A.K., Ekblom M., Karlsson A., Brøsen K. (2006). The Impact of CYP2C8 Polymorphism and Grapefruit Juice on the Pharmacokinetics of Repaglinide. Br. J. Clin. Pharmacol..

[192] Lilja J.J., Niemi M., Fredrikson H., Neuvonen P.J. (2007). Effects of Clarithromycin and Grapefruit Juice on the Pharmacokinetics of Glibenclamide. Br. J. Clin. Pharmacol..

[193] Owira P.M.O., Ojewole J.A.O. (2009). Grapefruit Juice Improves Glycemic Control but Exacerbates Metformin-Induced Lactic Acidosis in Non-Diabetic Rats. Methods Find. Exp. Clin. Pharmacol..

[194] Qurtam A.A., Mechchate H., Es-Safi I., Al-Zharani M., Nasr F.A., Noman O.M., Aleissa M., Imtara H., Aleissa A.M., Bouhrim M. (2021). Citrus Flavanone Narirutin, In Vitro and In Silico Mechanistic Antidiabetic Potential. Pharmaceutics.

[195] Cesar T.B., Ramos F.M.M., Ribeiro C.B. (2022). Nutraceutical Eriocitrin (Eriomin) Reduces Hyperglycemia by Increasing Glucagon-Like Peptide 1 and Downregulates Systemic Inflammation: A Crossover-Randomized Clinical Trial. J. Med. Food.

[196] Bertelli A., Biagi M., Corsini M., Baini G., Cappellucci G., Miraldi E. (2021). Polyphenols: From Theory to Practice. Foods.

[197] Walle T. (2007). Methoxylated Flavones, a Superior Cancer Chemopreventive Flavonoid Subclass?. Semin. Cancer Biol..

[198] Guo Y., Bruno R.S. (2015). Endogenous and Exogenous Mediators of Quercetin Bioavailability. J. Nutr. Biochem..

[199] Lim G.E., Li T., Buttar H.S. (2003). Interactions of Grapefruit Juice and Cardiovascular Medications: A Potential Risk of Toxicity. Exp. Clin. Cardiol..

[200] Zhou Y., Zheng J., Li Y., Xu D.-P., Li S., Chen Y.-M., Li H.-B. (2016). Natural Polyphenols for Prevention and Treatment of Cancer. Nutrients.

[201] Selma M.V., Espín J.C., Tomás-Barberán F.A. (2009). Interaction between Phenolics and Gut Microbiota: Role in Human Health. J. Agric. Food Chem..

[202] Galati G., O’Brien P.J. (2004). Potential Toxicity of Flavonoids and Other Dietary Phenolics: Significance for Their Chemopreventive and Anticancer Properties. Free Radic. Biol. Med..

[203] Komoroski B.J., Zhang S., Cai H., Hutzler J.M., Frye R., Tracy T.S., Strom S.C., Lehmann T., Ang C.Y.W., Cui Y.Y. (2004). Induction and Inhibition of Cytochromes P450 by the St. John’s Wort Constituent Hyperforin in Human Hepatocyte Cultures. Drug Metab. Dispos. Biol. Fate Chem..

[204] Awortwe C., Bruckmueller H., Cascorbi I. (2019). Interaction of Herbal Products with Prescribed Medications: A Systematic Review and Meta-Analysis. Pharmacol. Res..

[205] Nicolussi S., Drewe J., Butterweck V., Schwabedissen H.E.M. (2020). zu Clinical Relevance of St. John’s Wort Drug Interactions Revisited. Br. J. Pharmacol..

[206] Velingkar V.S., Gupta G.L., Hegde N.B. (2017). A Current Update on Phytochemistry, Pharmacology and Herb–Drug Interactions of *Hypericum perforatum*. Phytochem. Rev..

[207] Turton-Weeks S.M., Barone G.W., Gurley B.J., Ketel B.L., Lightfoot M.L., Abul-Ezz S.R. (2001). St John’s Wort: A Hidden Risk for Transplant Patients. Prog. Transplant. Aliso Viejo Calif.

[208] Martinho A., Silva S.M., Garcia S., Moreno I., Granadeiro L.B., Alves G., Duarte A.P., Domingues F., Silvestre S., Gallardo E. (2016). Effects of *Hypericum perforatum* Hydroalcoholic Extract, Hypericin, and Hyperforin on Cytotoxicity and CYP3A4 mRNA Expression in Hepatic Cell Lines: A Comparative Study. Med. Chem. Res..

[209] Smith P.F., Bullock J.M., Booker B.M., Haas C.E., Berenson C.S., Jusko W.J. (2004). Induction of Imatinib Metabolism by *Hypericum perforatum*. Blood.

[210] Ramsden D., Fullenwider C.L., Santos C., LeCluyse E.L. (2025). Quantitative Clinical Risk Assessment of CYP2C, UDP-Glucuronosyltransferase, P-Glycoprotein Induction, and Complex Drug-Drug Interactions Using TruVivo Human Hepatocyte Triculture Platform. Drug Metab. Dispos..

[211] Gümüs K.S., Teegelbekkers A., Sauter M., Meid A.D., Burhenne J., Weiss J., Blank A., Haefeli W.E., Czock D. (2024). Effect of Tacrolimus Formulation (Prolonged-Release vs. Immediate-Release) on Its Susceptibility to Drug-Drug Interactions with St. John’s Wort. Clin. Pharmacol. Drug Dev..

[212] Xie R., Tan L.H., Polasek E.C., Hong C., Teillol-Foo M., Gordi T., Sharma A., Nickens D.J., Arakawa T., Knuth D.W. (2005). CYP3A and P-Glycoprotein Activity Induction With St. John’s Wort in Healthy Volunteers From 6 Ethnic Populations. J. Clin. Pharmacol..

[213] Gödtel-Armbrust U., Metzger A., Kroll U., Kelber O., Wojnowski L. (2007). Variability in PXR-Mediated Induction of CYP3A4 by Commercial Preparations and Dry Extracts of St. John’s Wort. Naunyn. Schmiedebergs Arch. Pharmacol..

[214] Mueller S.C., Majcher-Peszynska J., Uehleke B., Klammt S., Mundkowski R.G., Miekisch W., Sievers H., Bauer S., Frank B., Kundt G. (2006). The Extent of Induction of CYP3A by St. John’s Wort Varies among Products and Is Linked to Hyperforin Dose. Eur. J. Clin. Pharmacol..

[215] Soleymani S., Bahramsoltani R., Rahimi R., Abdollahi M. (2017). Clinical Risks of St John’s Wort (*Hypericum perforatum*) Co-Administration. Expert Opin. Drug Metab. Toxicol..

[216] Borrelli F., Izzo A.A. (2009). Herb-Drug Interactions with St John’s Wort (*Hypericum perforatum*): An Update on Clinical Observations. AAPS J..

[217] Izzo A.A., Hoon-Kim S., Radhakrishnan R., Williamson E.M. (2016). A Critical Approach to Evaluating Clinical Efficacy, Adverse Events and Drug Interactions of Herbal Remedies. Phytother. Res..

[218] Chrubasik-Hausmann S., Vlachojannis J., McLachlan A.J. (2019). Understanding Drug Interactions with St John’s Wort (*Hypericum perforatum* L.): Impact of Hyperforin Content. J. Pharm. Pharmacol..

[219] Mannel M. (2004). Drug Interactions with St John’s Wort: Mechanisms and Clinical Implications. Drug Saf..

[220] Russo E., Scicchitano F., Whalley B.J., Mazzitello C., Ciriaco M., Esposito S., Patanè M., Upton R., Pugliese M., Chimirri S. (2014). *Hypericum perforatum*: Pharmacokinetic, Mechanism of Action, Tolerability, and Clinical Drug–Drug Interactions. Phytother. Res..

[221] Ehambarampillai D., Wan M.L.Y. (2025). A Comprehensive Review of Schisandra Chinensis Lignans: Pharmacokinetics, Pharmacological Mechanisms, and Future Prospects in Disease Prevention and Treatment. Chin. Med..

[222] Zhao J., Sun T., Wu J.-J., Cao Y.-F., Fang Z.-Z., Sun H.-Z., Zhu Z.-T., Yang K., Liu Y.-Z., Gonzalez F.J. (2017). Inhibition of Human CYP3A4 and CYP3A5 Enzymes by Gomisin C and Gomisin G, Two Lignan Analogs Derived from Schisandra Chinensis. Fitoterapia.

[223] Seo H.-J., Ji S.-B., Kim S.-E., Lee G.-M., Park S.-Y., Wu Z., Jang D.S., Liu K.-H. (2021). Inhibitory Effects of Schisandra Lignans on Cytochrome P450s and Uridine 5′-Diphospho-Glucuronosyl Transferases in Human Liver Microsomes. Pharmaceutics.

[224] Wan C.-K., Tse A.K., Yu Z.-L., Zhu G.-Y., Wang H., Fong D.W.F. (2010). Inhibition of Cytochrome P450 3A4 Activity by Schisandrol A and Gomisin A Isolated from Fructus Schisandrae Chinensis. Phytomedicine Int. J. Phytother. Phytopharm..

[225] Su L., Wang L., Li P., Mao C., Hao M., Lu T. (2017). Effects of Unprocessed Versus Vinegar-Processed Schisandra Chinensis on the Activity of CYP2A6, CYP2B6, CYP2C8, CYP2C9, CYP2C19 and CYP2D6 Enzymes in Rats. J. Anal. Bioanal. Tech..

[226] Unger M. (2013). Pharmacokinetic Drug Interactions Involving Ginkgo Biloba. Drug Metab. Rev..

[227] Robertson S.M., Davey R.T., Voell J., Formentini E., Alfaro R.M., Penzak S.R. (2008). Effect of Ginkgo Biloba Extract on Lopinavir, Midazolam and Fexofenadine Pharmacokinetics in Healthy Subjects. Curr. Med. Res. Opin..

[228] Uchida S., Yamada H., Xiao D.L., Maruyama S., Ohmori Y., Oki T., Watanabe H., Umegaki K., Ohashi K., Yamada S. (2006). Effects of Ginkgo Biloba Extract on Pharmacokinetics and Pharmacodynamics of Tolbutamide and Midazolam in Healthy Volunteers. J. Clin. Pharmacol..

[229] Zadoyan G., Rokitta D., Klement S., Dienel A., Hoerr R., Gramatté T., Fuhr U. (2012). Effect of Ginkgo Biloba Special Extract EGb 761^®^ on Human Cytochrome P450 Activity: A Cocktail Interaction Study in Healthy Volunteers. Eur. J. Clin. Pharmacol..

[230] Yan J., Gu Q., Meng C., Liu J., Liu F., Xia C. (2023). Panaxytriol Upregulates CYP3A4 Expression through the Interaction between Nuclear Regulators and DNA Response Elements. J. Ethnopharmacol..

[231] Wu J., Yao N., Hu Q., Liu M., Zhang H., Xiong Y., Hu J., Xia C. (2019). Effect of Panaxytriol on Cytochrome P450 3A4 via the Pregnane X Receptor Regulatory Pathway. Phytother. Res. PTR.

[232] Yang L., Wang Y., Xu H., Huang G., Zhang Z., Ma Z., Gao Y. (2019). Panax Ginseng Inhibits Metabolism of Diester Alkaloids by Downregulating CYP3A4 Enzyme Activity via the Pregnane X Receptor. Evid.-Based Complement. Altern. Med. ECAM.

[233] Anderson G.D., Rosito G., Mohustsy M.A., Elmer G.W. (2003). Drug Interaction Potential of Soy Extract and Panax Ginseng. J. Clin. Pharmacol..

[234] Zhang L., Yan J., Liu J., Meng C., Liu F., Xia C. (2022). Panaxytriol Upregulates CYP3A4 Expression Based on the Interaction of PXR, CAR, HSP90α, and RXRα. Phytomedicine Int. J. Phytother. Phytopharm..

[235] Elens L., van Gelder T., Hesselink D.A., Haufroid V., van Schaik R.H. (2013). CYP3A4*22: Promising Newly Identified CYP3A4 Variant Allele for Personalizing Pharmacotherapy. Pharmacogenomics.

[236] Zhang Y., Wang Z., Wang Y., Jin W., Zhang Z., Jin L., Qian J., Zheng L. (2024). CYP3A4 and CYP3A5: The Crucial Roles in Clinical Drug Metabolism and the Significant Implications of Genetic Polymorphisms. PeerJ.

[237] Wang D., Guo Y., Wrighton S.A., Cooke G.E., Sadee W. (2011). Intronic Polymorphism in CYP3A4 Affects Hepatic Expression and Response to Statin Drugs. Pharmacogenom. J..

[238] Elalem E.G., Jelani M., Khedr A., Ahmad A., Alaama T.Y., Alaama M.N., Al-Kreathy H.M., Damanhouri Z.A. (2022). Association of Cytochromes P450 3A4*22 and 3A5*3 Genotypes and Polymorphism with Response to Simvastatin in Hypercholesterolemia Patients. PLoS ONE.

[239] Jansen M.E., Rigter T., Rodenburg W., Fleur T.M.C., Houwink E.J.F., Weda M., Cornel M.C. (2017). Review of the Reported Measures of Clinical Validity and Clinical Utility as Arguments for the Implementation of Pharmacogenetic Testing: A Case Study of Statin-Induced Muscle Toxicity. Front. Pharmacol..

[240] Gao Y., Zhang L., Fu Q. (2008). CYP3A4*1G Polymorphism Is Associated with Lipid-Lowering Efficacy of Atorvastatin but Not of Simvastatin. Eur. J. Clin. Pharmacol..

[241] Abdullah-Koolmees H., van Keulen A.M., Nijenhuis M., Deneer V.H.M. (2021). Pharmacogenetics Guidelines: Overview and Comparison of the DPWG, CPIC, CPNDS, and RNPGx Guidelines. Front. Pharmacol..

[242] Kaye J.B., Schultz L.E., Steiner H.E., Kittles R.A., Cavallari L.H., Karnes J.H. (2017). Warfarin Pharmacogenomics in Diverse Populations. Pharmacother. J. Hum. Pharmacol. Drug Ther..

[243] Hirota T., Fujita Y., Ieiri I. (2020). An Updated Review of Pharmacokinetic Drug Interactions and Pharmacogenetics of Statins. Expert Opin. Drug Metab. Toxicol..

[244] Adachi K., Ohyama K., Tanaka Y., Sato T., Murayama N., Shimizu M., Saito Y., Yamazaki H. (2023). High Hepatic and Plasma Exposures of Atorvastatin in Subjects Harboring Impaired Cytochrome *P450 3A4∗16* Modeled after Virtual Administrations and Possibly Associated with Statin Intolerance Found in the Japanese Adverse Drug Event Report Database. Drug Metab. Pharmacokinet..

[245] Saiz-Rodríguez M., Almenara S., Navares-Gómez M., Ochoa D., Román M., Zubiaur P., Koller D., Santos M., Mejía G., Borobia A.M. (2020). Effect of the Most Relevant CYP3A4 and CYP3A5 Polymorphisms on the Pharmacokinetic Parameters of 10 CYP3A Substrates. Biomedicines.

[246] Jordan I.K., Sharma S., Mariño-Ramírez L. (2023). Population Pharmacogenomics for Health Equity. Genes.

[247] Alvarado A.T., Muñoz A.M., Ybañez-Julca R.O., Pineda-Pérez M., Tasayco-Yataco N., Bendezú M.R., García J.A., Surco-Laos F., Chávez H., Laos-Anchante D. (2023). SLCO1B1 and CYP3A4 Allelic Variants Associated with Pharmacokinetic Interactions and Adverse Reactions Induced by Simvastatin and Atorvastatin Used in Peru: Clinical Implications. J. Pharm. Pharmacogn. Res..

[248] Swen J.J., van der Wouden C.H., Manson L.E., Abdullah-Koolmees H., Blagec K., Blagus T., Böhringer S., Cambon-Thomsen A., Cecchin E., Cheung K.-C. (2023). A 12-Gene Pharmacogenetic Panel to Prevent Adverse Drug Reactions: An Open-Label, Multicentre, Controlled, Cluster-Randomised Crossover Implementation Study. Lancet.

[249] Brunham L.R., Baker S., Mammen A., Mancini G.B.J., Rosenson R.S. (2018). Role of Genetics in the Prediction of Statin-Associated Muscle Symptoms and Optimization of Statin Use and Adherence. Cardiovasc. Res..

[250] Sivadas A., Sahana S., Jolly B., Bhoyar R.C., Jain A., Sharma D., Imran M., Senthivel V., Divakar M.K., Mishra A. (2024). Landscape of Pharmacogenetic Variants Associated with Non-Insulin Antidiabetic Drugs in the Indian Population. BMJ Open Diabetes Res. Care.

[251] Silva P., Jacobs D., Kriak J., Abu-Baker A., Udeani G., Neal G., Ramos K. (2021). Implementation of Pharmacogenomics and Artificial Intelligence Tools for Chronic Disease Management in Primary Care Setting. J. Pers. Med..

[252] Tipnoppanon S., Udomnilobol U., Siwamogsatham S., Vorasettakarnkij Y., Sukasem C., Prueksaritanont T., Chariyavilaskul P., Yodsurang V., Srimatimanon T., Chamnanphon M. (2025). Impacts of Pharmacokinetic Gene Polymorphisms on Steady-State Plasma Concentrations of Simvastatin in Thai Population. Clin. Transl. Sci..

[253] Ulvestad M., Skottheim I.B., Jakobsen G.S., Bremer S., Molden E., Åsberg A., Hjelmesæth J., Andersson T.B., Sandbu R., Christensen H. (2013). Impact of OATP1B1, MDR1, and CYP3A4 Expression in Liver and Intestine on Interpatient Pharmacokinetic Variability of Atorvastatin in Obese Subjects. Clin. Pharmacol. Ther..

[254] Li H., He J., Jia W. (2016). The Influence of Gut Microbiota on Drug Metabolism and Toxicity. Expert Opin. Drug Metab. Toxicol..

[255] Spanogiannopoulos P., Bess E.N., Carmody R.N., Turnbaugh P.J. (2016). The Microbial Pharmacists within Us: A Metagenomic View of Xenobiotic Metabolism. Nat. Rev. Microbiol..

[256] Wilson I.D., Nicholson J.K. (2017). Gut Microbiome Interactions with Drug Metabolism, Efficacy, and Toxicity. Transl. Res. J. Lab. Clin. Med..

[257] Togao M., Kawakami K., Otsuka J., Wagai G., Ohta-Takada Y., Kado S. (2020). Effects of Gut Microbiota on In Vivo Metabolism and Tissue Accumulation of Cytochrome P450 3A Metabolized Drug: Midazolam. Biopharm. Drug Dispos..

[258] Collins S.L., Patterson A.D. (2020). The Gut Microbiome: An Orchestrator of Xenobiotic Metabolism. Acta Pharm. Sin. B.

[259] Enright E.F., Gahan C.G.M., Joyce S.A., Griffin B.T. (2016). The Impact of the Gut Microbiota on Drug Metabolism and Clinical Outcome. Yale J. Biol. Med..

[260] Yang H., Zhang Y., Zhou R., Wu T., Zhu P., Liu Y., Zhou J., Xiong Y., Xiong Y., Zhou H. (2023). Antibiotics-Induced Depletion of Rat Microbiota Induces Changes in the Expression of Host Drug-Processing Genes and Pharmacokinetic Behaviors of CYPs Probe Drugs. Drug Metab. Dispos..

